# An Automated Micro‐Immunobeads‐Based Electromagnetic Operation System (MEMOs) for Blood Testing of Alzheimer's Disease

**DOI:** 10.1002/advs.202509376

**Published:** 2025-08-18

**Authors:** Xiaoxue Fan, Xinyu Ye, Jiangrui Xu, Tianjiao Mao, Ruotong Zhang, Xinyuan Zhou, Christina C. K. Au Yeung, Chang Li, Raymond Chuen‐Chung Chang, Haisong Lin, Ho Cheung Shum

**Affiliations:** ^1^ Department of Mechanical Engineering The University of Hong Kong Pokfulam Road Hong Kong SAR 000000 China; ^2^ Laboratory of Neurodegenerative Diseases, School of Biomedical Sciences, LKS Faculty of Medicine The University of Hong Kong Laboratory Block, Rm. L4‐49, 21 Sassoon Road, Pokfulam Hong Kong SAR 000000 China; ^3^ School of Engineering Westlake University 600 Dunyu Road Hangzhou Zhejiang 310030 China; ^4^ Advanced Biomedical Instrumentation Centre Hong Kong Science Park Shatin, New Territories Hong Kong SAR 000000 China; ^5^ Research Center for Industries of the Future Westlake University 600 Dunyu Road Hangzhou Zhejiang 310030 China; ^6^ Department of Chemistry and Department of Biomedical Engineering City University of Hong Kong Hong Kong SAR 000000 China

**Keywords:** automation, diagnosis, electromagnetic, microfluidics, point‐of‐care

## Abstract

The anticipated rise in the prevalence of Alzheimer's disease (AD) under global demographic shifts necessitates the development of universally accessible screening and diagnostic tools, of which blood tests hold the promise. However, the widespread adoption of blood tests remains impeded by the lack of analytically validated biomarkers and accessible decentralized platforms. Here, the study presents a portable and automated micro‐immunobeads‐based electromagnetic operation system, termed MEMOs, targeting potential AD blood biomarker exosome‐bound Aβ42 (Exo‐Aβ42), for automated blood testing of AD. MEMOs integrates a microfluidic exosome‐detection biological chip (μ‐Exo Biochip) embedded with a magnetic field‐responsive assay for detecting Exo‐Aβ42 and a microbead operation electromagnetic chip (μ‐Bead EMchip) for programmable and addressable actuation, collectively delivering a high level of accurate and automated AD testing. In both animal and clinical samples, the levels of Exo‐Aβ42 measured using MEMOs have shown significant correlations with various progressive stages of AD. With the features of cost‐effective manufacturing, minimized hands‐on time, and an easy operation workflow, MEMOs offers an accessible pathway toward diagnosing and monitoring the progression of AD in decentralized practice.

## Graphical abstract

This work presents an automated and accessible point‐of‐care device, MEMOs, for the detection of exosomal Aβ42 protein, a potential blood biomarker for Alzheimer’s disease. MEMOs, with its automated operation workflow, minimizes hands‐on time and maintains high sensitivity in diagnosing AD, allowing non‐specialists to conduct tests at home and offering a more accessible solution for testing in decentralized settings.

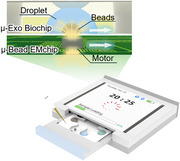

## 1 Introduction

The growing trend of the aging population has brought new challenges to global health and social systems. This demographic shift necessitates preemptive planning in public health, particularly in addressing age‐related diseases, such as Alzheimer's disease (AD).^[^ ^1^, ^2^ ^]^ As the most common cause of dementia, AD is a progressive and irreversible neurological disorder that leads to a decline in both mental and physical abilities, severely interfering with normal life.^[^ ^3^, ^4^, ^5^ ^]^ With the aging population, the incidence and prevalence of AD are expected to rise significantly, imposing a substantial burden on healthcare systems, caregivers, and society.^[^ ^2^, ^3^, ^6^, ^7^, ^8^ ^]^ Consequently, there is a growing need for early diagnostic tools to provide timely interventions and automated screening programs for the rising elderly population, addressing the impact of AD as an emerging worldwide health issue.^[^ ^9^, ^10^, ^11^, ^12^ ^]^

Current AD diagnostic standards depend on clinical evaluations^[^ ^13^, ^14^, ^15^ ^]^ and neuroimaging techniques,^[^ ^16^, ^17^, ^18^ ^]^ which lack objectivity for reliable assessments^[^ ^19^, ^20^, ^21^ ^]^ and accessibility for broader applications,^[^ ^16^, ^18^, ^22^ ^]^ respectively, thereby limiting their effectiveness in screening. The existing molecular assay for cerebrospinal fluid testing is highly invasive and carries the risk of complications, rendering it unsuitable as an early diagnostic tool.^[^ ^23^, ^24^ ^]^ To address existing limitations, blood testing emerges as a promising area of research that has the potential to expand AD detection capacities and facilitate large‐scale screening efforts.^[^ ^3^, ^25^, ^26^, ^27^, ^28^, ^29^ ^]^

Despite recent advances, two major challenges remain in blood testing for AD. First, the use of amyloid beta protein (Aβ), a core biomarker for AD, in blood for diagnostic purposes remains under evaluation as numerous contradictory findings have been reported in prior studies.^[^ ^30^, ^31^, ^32^ ^]^ More specifically, the lack of correlation between blood Aβ levels and neuroimaging findings challenges its clinical utility.^[^ ^33^, ^34^, ^35^ ^]^ Second, there is a shortage of appropriate automated screening devices to facilitate blood testing for AD.^[^ ^32^, ^36^, ^37^, ^38^ ^]^ The enzyme‐linked immunosorbent assay, the most prevalent immunological detection tool, is time‐consuming, labor‐intensive, and requires skilled personnel due to its complex manual steps.^[^ ^39^ ^]^ Although advances in digital microfluidic bioassays^[^ ^40^, ^41^, ^42^ ^]^ and nanoprobe‐enabled lateral flow immunoassays^[^ ^43^, ^44^ ^]^ have led to a reduction of manual procedures and an expansion of the scope of applications, the digital microfluidic bioassays encounter biocompatibility issues, while the lateral flow immunoassays face challenges of limited sensitivity. Alternative electrochemical tests, such as field‐effect transistors,^[^ ^45^ ^]^ and optical spectroscopy measurements, for instance, surface‐enhanced Raman spectroscopy,^[^ ^46^ ^]^ require specialized instruments and specific environmental conditions for analysis, limiting their feasibility for use outside laboratory settings.^[^ ^47^ ^]^

**Figure 1:**
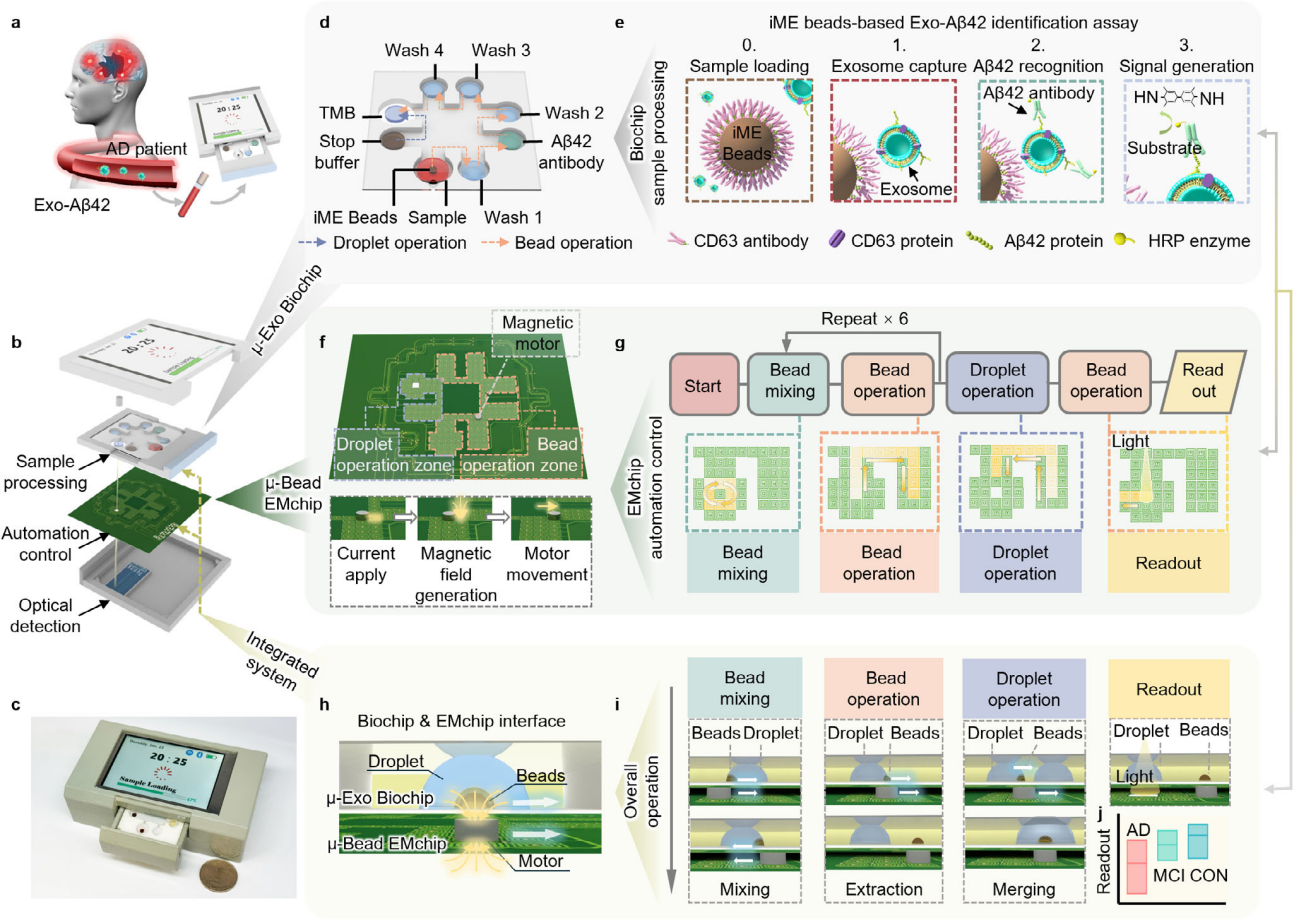
Schematic illustration of the MEMOs. a) Schematic overview of using MEMOs for Alzheimer's disease (AD) diagnosis by detecting Exo‐Aβ42 in plasma. b) Exploded‐view illustration of the main components of the MEMOs, which includes a sample processing microfluidic exosome‐detection biological chip (μ‐Exo Biochip), a microbead operation electromagnetic chip (μ‐Bead EMchip), and an optical signal detection module. c) Optical image of a representative MEMOs operating in the sample loading state. One Hong Kong dollar coin used for scale. d) The μ‐Exo Biochip incorporates an iME beads‐based Exo‐Aβ42 identification assay (iME beads and reagents) designed for the detection of Exo‐Aβ42. e) The mechanism of the iME beads‐based Exo‐Aβ42 identification assay on the μ‐Exo Biochip, which involves first identifying the exosome membrane endogenous protein CD63, subsequently recognizing the exosome membrane‐bound exogenous protein Aβ42, and finally generating signals. f) The μ‐Bead EMchip in MEMOs facilitates automated sample processing, which involves both droplet operation and bead operation. Inset: The step‐motion mechanism of a magnetic motor, which begins with a programmed current applied to a specific coil, followed by the generation of a magnetic field, and ultimately driving the motor in the direction of the induced magnetic field. g) Overall workflow of the μ‐Bead EMchip for automation control. The yellow array indicates the programmed navigation paths of the magnetic motor to execute various bead and droplet motion commands. h) Cross‐sectional illustration showing the interface between the integrated μ‐Exo Biochip and μ‐Bead EMchip. The magnetic motor is controlled by the μ‐Bead EMchip through programmed magnetic field induction, triggering the motions of beads and droplets in the μ‐Exo Biochip to complete the iME beads‐based Exo‐Aβ42 identification assay procedure. i) Overall illustration of the integrated system capable of performing diverse functionalities, including bead mixing, bead extraction, droplet merging, and readout. j) The optical readouts of the Exo‐Aβ42 signal detected by MEMOs are utilized to distinguish Alzheimer's disease (AD), mild cognitive impairment (MCI), and healthy controls (CON).

To broaden the accessibility of AD blood testing, here we develop an automated micro‐immunobeads‐based electromagnetic operation system, termed MEMOs, targeting blood exosome‐bound Aβ42 (Exo‐Aβ42) analysis for automatic testing of AD (**Figure** **1**a). Instead of using the controversial total‐circulating Aβ42 protein, our study employs neuron‐derived exosome‐bound Aβ42, which has been shown to reflect brain plaque deposition, thereby serving as a potential indicator for AD blood testing.^[^ ^48^, ^49^, ^50^, ^51^ ^]^ To achieve an automated detection system, MEMOs is designed with the comprehensive internal integration of both a microfluidic exosome‐detection biological chip (μ‐Exo Biochip) and a microbead operation electromagnetic chip (μ‐Bead EMchip) (Figure 1b), along with an external, vest‐pocket‐sized user‐friendly interface (Figure 1c). The μ‐Exo Biochip (Figure 1d) incorporates our developed automation‐compatible immune magnetic exosome beads (iME beads)‐based Exo‐Aβ42 identification assay (Figure 1e) for sample extraction, biomarker recognition, and signal amplification. The μ‐Bead EMchip (Figure 1f) generates localized addressable magnetic fields to actuate magnet motors robustly and is designed with a specific pathway to perform programmable sequences of movement automatically (Figure 1g). The integrated system is assembled in a vertical stack, with the μ‐Exo Biochip positioned atop the μ‐Bead EMchip (Figure 1h), allowing contactless guidance and precise control of the beads and droplets. This configuration facilitates the implementation of diverse functionalities, ultimately leading to the automation of the entire detection assay procedures (Figure 1i). Through testing with MEMOs, we demonstrate that plasma Exo‐Aβ42 levels are correlated and distinguishable among individuals at various stages of AD progression, including AD, mid cognitive impairment (MCI), and control group (CON) (Figure 1j). These findings underscore the potential of MEMOs as an accessible and automated diagnostic tool for AD.

## 2 Results

### 2.1 MEMOs Assay Development and Characterization

To detect the target biomolecule Exo‐Aβ42 with high sensitivity and automated processing, we developed an iME beads‐based Exo‐Aβ42 identification assay with dual marker sites targeting capability to specifically identify both the exosomal marker and the amyloid beta marker, respectively. Magnetic microbeads were selected as the solid substrate to provide the assay with magnetic field‐responsive properties, enabling subsequent downstream isolation, extraction, and transportation between multiphase interfaces.^[^ ^52^, ^53^ ^]^ The use of magnetic microbeads also enhanced the availability of immobilization sites for biomolecules because of their extensive surface area.^[^ ^54^, ^55^ ^]^ A sequence of assay processing steps was meticulously developed and systematically evaluated to achieve target molecule identification, consisting of iME beads development, exosome capture, and Aβ42 recognition and signal generation.

**Figure 2:**
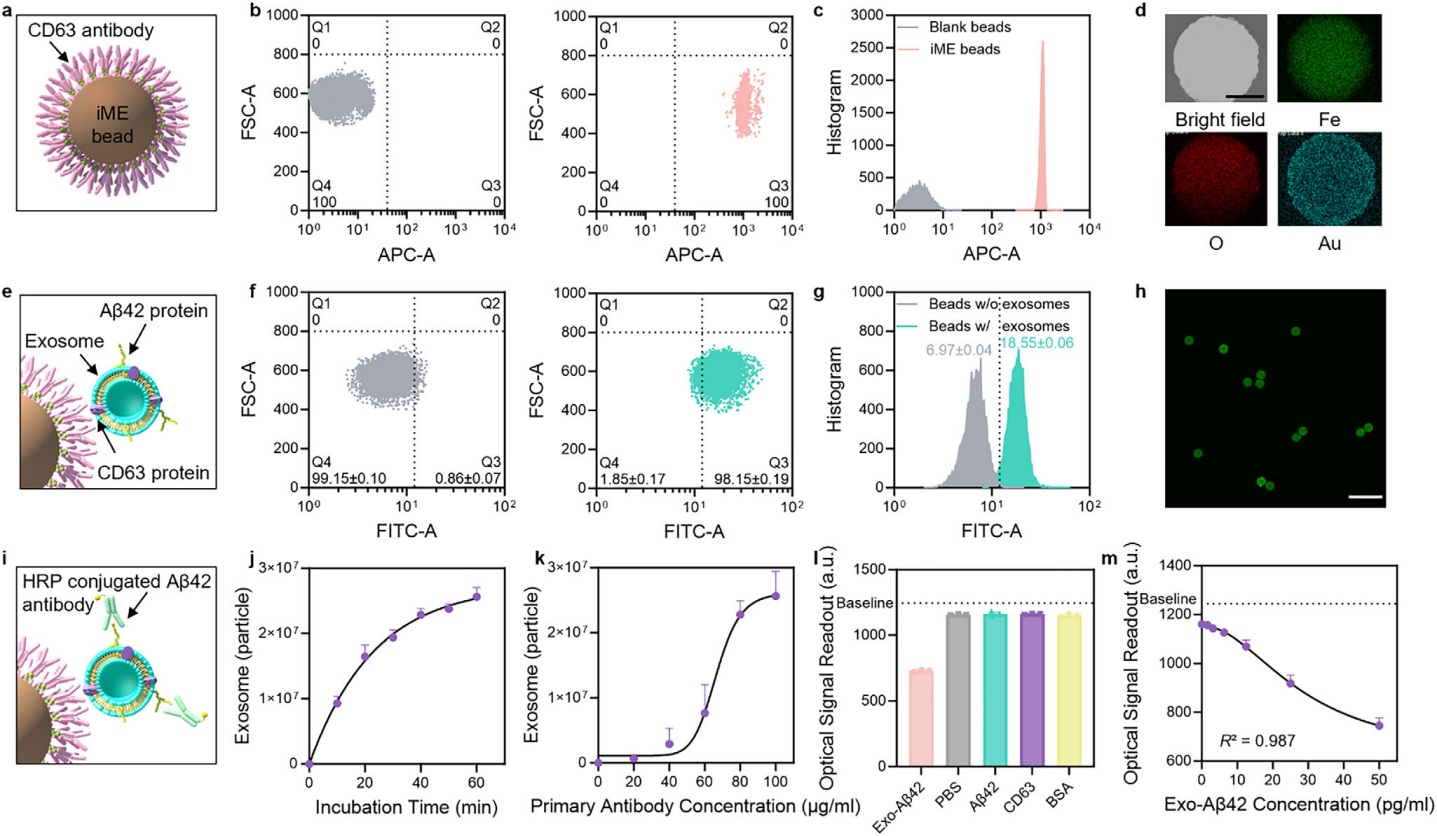
Characterization of the capture efficiency and sensing performance of the iME beads‐based Exo‐Aβ42 identification assay. a) Schematic illustration of the functionalized immune magnetic exosome beads (iME beads). Anti‐CD63 primary antibody (pink) is coated on the surface of the beads. b) Flow cytometry dot‐plot analysis of control beads (left) and iME beads (right). APC dye was used to target the antibody for signaling. c) Histogram of the APC signal intensities for blank beads and iME beads. d) SEM‐EDS analysis elemental mapping (Fe ‐ green, O ‐ red, Au ‐ blue) of iME beads. Gold nanoparticles (AuNPs) were conjugated to the antibody for visualization. Scale bar = 1 µm. e) The process of identifying and capturing the exosome membrane endogenous protein CD63 in the iME beads‐based Exo‐Aβ42 identification assay. f) Flow cytometry dot‐plot analysis of single blank iME beads (left) and beads captured with exosomes (right). FITC dye was used to label exosomes. g) Histogram of the FITC signal intensities of the blank iME beads and the beads captured with exosomes. Error bars indicate SDs (*n* = 3). h) Fluorescent microscope image of iME beads captured with FITC‐labeled exosomes (after extraction). Scale bar = 10 µm. i) The process of recognizing and signaling the exosome membrane‐bound exogenous protein Aβ42 in the iME beads‐based Exo‐Aβ42 identification assay. j) Optimization of the iME beads exosome capture quantity as a function of incubation time. Error bars indicate SDs (*n* = 3). k) Optimization of iME beads exosome capture quantity as a function of the concentration of primary antibody. Error bars indicate SDs (*n* = 3). l) The specificity of the iME beads‐based Exo‐Aβ42 identification assay, showing the sensing signals of the target Exo‐Aβ42 protein and three non‐target proteins (Aβ42, CD63, and BSA). Error bars indicate SDs (*n* = 3). The baseline indicates the background signal of the optical detection sensor. m) The calibration plot of the response of the iME beads‐based Exo‐Aβ42 identification assay with 0–50 pg ml^−1^ Exo‐Aβ42 (Four‐parameter logistic fitting, *R* ^2^ = 0.987). Error bars indicate SDs (*n* = 3). The baseline indicates the background signal of the optical detection sensor.

To develop iME beads with high selective isolation capability, the typical exosome marker CD63 was targeted (**Figure** **2**a; Figure S1a, Supporting Information). CD63, a tetraspanin protein that is highly concentrated in the exosomal membrane, is widely utilized in the immunoisolation of exosomes.^[^ ^45^, ^56^, ^57^ ^]^ Biotinylated anti‐CD63 antibodies were conjugated to the surface of streptavidin‐positive magnetic beads via non‐covalent interactions for the purpose of exosome identification and enrichment. The immunological functionalization efficiency at the single bead level was characterized by flow cytometry (Figure 2b,c; Figure S1b, Supporting Information). Allophycocyanin (APC) fluorescence dye was used to label the CD63 antibody, thereby facilitating the characterization of functional efficiency. After gating, the quartile distribution within the dot plot, based on the fluorescent intensity of individual beads, showed that blank beads were primarily distributed in the fourth quadrant Q4, while iME beads were dispersed in the third quadrant Q3. Compared to blank beads, the APC fluorescence signal of the iME beads shifted significantly, whereas the forward scattering (FSC) signal remained unchanged. The APC signal shift confirmed effective antibody immobilization on individual beads, while the unchangeable FSC signal indicated that the immunological coating did not cause detectable size changes of the beads. The histogram of the APC signal for single beads showed a narrow peak width for the iME beads group, indicating the homogeneity of immunological functionalization. No cross‐reaction was detected between blank streptavidin beads and APC antibody staining (Figure S1c–h, Supporting Information). The functionalization performance was further confirmed by scanning electron microscope (SEM), where an AuNPs‐conjugated secondary antibody was used to mark the surface functionalized sites of the iME beads. The results showed that AuNPs were uniformly localized on the surface of the iME beads, indicating the successful conjugation of the immune coating (Figure S2, Supporting Information). Energy dispersive X‐ray spectroscopy obtained via SEM demonstrated that the Au element was uniformly distributed and co‐localized with the Fe and O elements on the beads surface (Figure 2d), whereas in the control group, the Au element was not detected (Figure S3, Supporting Information).

To evaluate the exosome immunocapture performance of iME beads, exosomes isolated from the human neuroblastoma cell line SH‐SY5Y (a common cell model used to study molecular events leading to AD^[^ ^58^ ^]^), were employed for in vitro studies (Figure 2e). The isolated vesicles exhibited a characteristic exosomal cup‐shaped morphology under transmission electron microscopy (TEM) (Figure S4a, Supporting Information) and displayed a uniform distribution of particle sizes, with an average diameter of ≈150 nm, as measured by nanoparticle tracking analysis (NTA) (Figure S4b,c, Supporting Information). Western blotting confirmed that the exosome‐specific protein markers, including our target CD63 protein and the reference TSG101 protein, were prominently expressed in the isolated vesicles (Figure S4d, Supporting Information). The performance of exosome capture was investigated through flow cytometry (Figure 2f,g; Figure S5, Supporting Information) using FITC exosome staining. The quartile distribution showed that 99.15 ± 0.10% of iME beads before exosome capture were distributed in the Q4 zone, while after incubation with exosomes, 98.15 ± 0.19% of the beads shifted to the Q3 zone, indicating the effectiveness of exosome capture. The histogram of the FITC signal for individual beads showed that the mean fluorescence intensity increased from 6.97 ± 0.04 to 18.55 ± 0.06, further suggesting the immune affinity of iME beads for exosome isolation. Confocal microscope images displayed a uniform FITC signal at the surface of the exosome‐captured beads (Figure 2h) and no FITC signal in the control group (Figure S6, Supporting Information), indicating the uniformity of the capture capability of the iME beads.

To process Aβ42 recognition and enzymatic signal generation procedures, a horseradish peroxidase (HRP) enzyme‐conjugated capture antibody was prepared to target the second marker site, the Aβ42 protein (Figure 2i). Its substrate 3,3’‐diaminobenzidine tetrahydrochloride (TMB) was used to amplify the optical signal. To facilitate in vitro experiments, the exosome‐amyloid beta associations were prepared by self‐assembling Aβ42 aggregates onto the membranes of exosomes (Figure S7a, Supporting Information). The morphology of these associations was confirmed through transmission electron microscopy and scanning transmission electron microscopy (Figure S7b,c, Supporting Information). Notably, both the Exo‐Aβ42 and free Aβ42 aggregates co‐existed in the prepared sample, implying that Exo‐Aβ42 represented a subpopulation within the total Aβ42 environment. Using the prepared Exo‐Aβ42 biomarkers, we systematically examined the essential parameters for the exosome capture process, including incubation times (Figure 2j) and immune functionalization concentrations (Figure 2k), which were quantified with the assistance of a commercial exosome quantification kit (Figure S8, Supporting Information). Meanwhile, the enzymatic signal generation process was also analyzed to maximize the signal‐to‐noise ratio through modifications of blocking buffer (PBS, PBST, and Block ACE buffer), washing buffer (PBS, PBST, and Block ACE buffer), enzyme concentration (500x, 1000x, and 2000x), the addition of immune affinity enhancer (adding and non‐adding), and wash time (Figure S9, Supporting Information).

After successfully developing the iME beads‐based Exo‐Aβ42 identification assay, the entire assay was tested for specificity and sensitivity on‐chip (Figure 1d). A significant signal was observed upon introducing the target molecule Exo‐Aβ42, compared to three non‐target proteins (Aβ42, CD63, and BSA) (Figure 2l). It was worth noting that the amyloid beta protein Aβ42 and the exosomal marker protein CD63 did not generate any background noise, indicating that free Aβ42 protein and exosomes did not affect the detection specificity of our assay. These results further confirmed the dual sites targeting mechanism of our iME beads‐based Exo‐Aβ42 identification assay. After establishing specificity, the sensing behavior of the assay was investigated by spiking the prepared exosome‐amyloid beta associations in PBS and measuring the results on‐chip (Figure S10, Supporting Information). Based on the quantified results, the limit of detection of our platform was at 2.16 pg ml^−1^ with a regression parameter *R* ^2^ = 0.987 (Figure 2m). This sensitivity covers the reported concentration range of human plasma Aβ42 protein.^[^ ^59^, ^60^, ^61^ ^]^

### 2.2 MEMOs Automation and Integration

To achieve fully automated immunosensing operations without requiring user intervention, MEMOs was designed based on a vertical stack assembly, comprising 1) a μ‐Exo Biochip embedded with target molecule Exo‐Aβ42 identification assay, 2) a μ‐Bead EMchip equipped with programmable and addressable motors, and 3) an optical signal sensing model for signal readout (Figure 1b).

**Figure 3:**
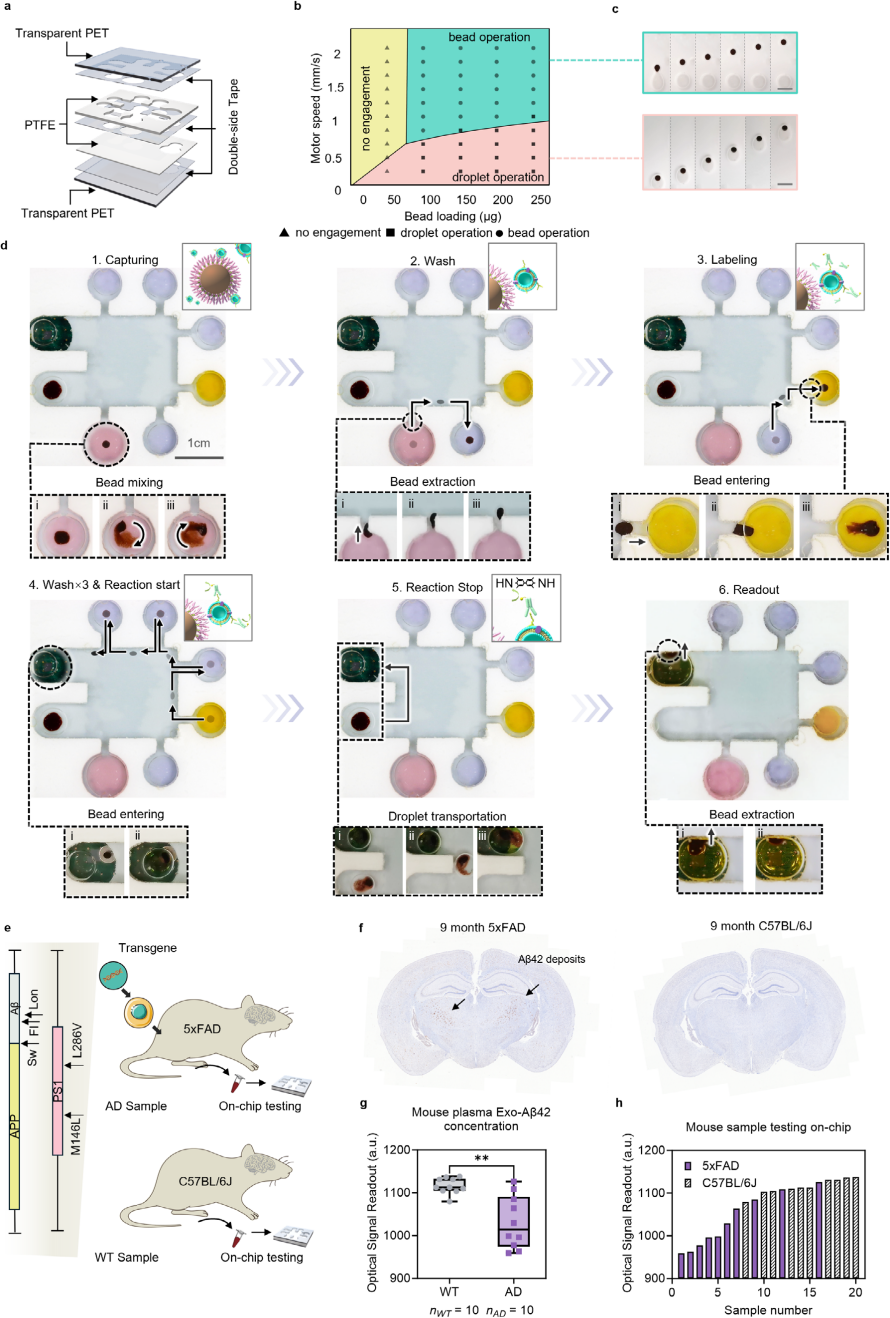
An automation and validation of the developed assay on‐chip. a) Layer design of the μ‐Exo Biochip. b) Analytical (lines) and experimental (dots) operation zones for beads and droplets based on adjusted bead loading mass and programmed magnet motor speeds. c) Sequential optical images depicting bead operation (extraction of beads, Δ*t* = 150 ms, top) and droplet operation (transportation of droplets, Δ*t* = 500 ms, bottom). Scale bar = 2 mm. d) Sequential optical images showcasing the automation of the iME beads‐based Exo‐Aβ42 identification assay on‐chip. The bottom section features a zoomed‐in view of the actuation details, while the top right corner depicts the corresponding assay step. e) Demonstration of the animal samples tested on‐chip (Alzheimer's mouse model: 5xFAD and wild‐type mouse control: C57BL/6J). The 5xFAD Alzheimer's mouse model is transgenic for five AD‐linked gene mutations. f) Immunohistochemical (IHC) staining of Aβ42 aggregates in the hippocampus of 5xFAD (left) and C57BL/6J (right) mice. Dark plaques indicated Aβ42 deposits. g,h) Validation of the detection performance of MEMOs for sensing Exo‐Aβ42 levels in AD mice (*n* = 10) and wild‐type mice plasma (*n* = 10). g) Box plots and h) waterfall plots showing the optical signal readout by MEMOs. Statistical analysis was done using unpaired Mann–Whitney nonparametric test. *P < 0.05, **P < 0.01, ***P < 0.00, ****P < 0.0001.

The μ‐Exo Biochip consisted of reagent reservoirs for encapsulating various assay reagents, an oil zone for reagents isolation and beads/droplets transportation, and a detection chamber for signal readout (Figure 1d). The structure of the μ‐Exo Biochip was laser‐engraved on a polytetrafluoroethylene (PTFE) substrate due to its excellent resistance to molecular adsorption,^[^ ^62^ ^]^ which helped prevent assay reagent residues, as well as its non‐wetting property (Figure S11, Supporting Information), which facilitated the manipulation of beads and droplets. Transparent polyethylene terephthalate (PET) sheets were utilized to create a sealed environment for the assay reagents and to form a transparent detection region for signal readout. Different layers were assembled using patterned double‐sided tapes (**Figure** **3**a).

To implement our developed iME beads‐based Exo‐Aβ42 identification assay on the μ‐Exo Biochip in a fully automated manner, it was essential to thoroughly investigate the key actuation conditions that govern the operation behaviors and movement dynamics of beads and droplets^[^ ^52^, ^63^, ^64^ ^]^ (Note S1, Supporting Information). The entire system was analyzed with the magnetic force from the magnet motor, serving as the external driving force, and the friction force from the sliding contact surfaces, acting as the opposing external resistance force. The strength of the magnetic force was proportional to the bead loading mass, as the motor‐generated magnetic field exerted a direct influence on the bead cluster. The friction force was proportional to the transport velocity of the droplet, which can be regulated by the motor movement speed in our system.

To differentiate various motion conditions, it was essential to consider the internal force between beads and droplet. The internal force, resulting from the deformation of the droplet surface, was related to the deformed curvature length of the droplet. Therefore, the maximum internal force can be estimated as the entire circumference of the bead cluster contributed to the deformation of the surface, making it proportional to the cube root of the bead loading mass.^[^ ^63^ ^]^ In bead operation, the magnetic force served as the driving force, while the internal force acted as the resistance force. In droplet operation, the internal force served as the driving force, while the friction force acted as the resistance force. Therefore, to facilitate bead operation, we can increase the bead loading mass to enhance the magnetic force. To enable more effective droplet operation, we can reduce the actuation velocity and thus decrease the friction force.

By systematically balancing the three forces present in our system, we analytically built a model to explore the mechanisms underlying the manipulation and interaction between bead and droplet operations (Figure 3b, **line**). The complete derivation process was shown in Note S1 (Supporting Information). The modelling results showed three different zones based on different programmed motor speeds and bead loading masses. In the no engagement zone (yellow), the magnetic force was insufficient to overcome either the bead resistance internal force or the droplet friction force, rendering the motor movement ineffective for driving neither bead nor droplet motions. In the droplet operation zone (pink), for the bead, the magnetic force equalled to the internal force, while the internal force had not yet reached its maximum at this moment, making it ineffective for bead extraction from the droplet; for the droplet, the internal force was equal to the friction force, resulting in steady droplet movement. In the bead operation zone (lake blue), for the bead, the magnetic force exceeded the maximum of the internal force, allowing the bead cluster to be extracted from the droplet; for the droplet, the internal force did not exceed the friction force, preventing the motor from operating the droplet.

We also conducted experiments to confirm conclusions drawn from the analytical model. The results demonstrated three different operation conditions that aligned with the analytical operation diagram (Figure 3b, **dots**). The sequential optical images corresponding to two typical motion zones were shown in Figure 3c. Under droplet operation conditions (Figure 3c, **bottom**), both the beads and the droplet could steadily follow the movement of the motor. Under bead operation conditions (Figure 3c, **top**), the bead cluster could be extracted and consistently followed the actuation of the motor, whereas the droplet was left behind. Based on the analytical and experimental investigations, we found that adjusting the bead loading mass or regulating the motor speed can effectively switch between bead operation and droplet operation modes, providing flexibility in automating actuation functionalities and biochemical reactions within our system.

The μ‐Bead EMchip consisted of a programmable navigation matrix comprising electromagnetic coil elements and magnet motors with the comparable size of the coil unit^[^ ^65^, ^66^ ^]^ (Figure 1f). The coil elements could be programmed and sequentially activated to generate temporary magnetic fields when the current was applied to the adjacent coil elements. Following the guidance of the successively generated magnetic field, the motor can execute a series of consecutive functionalities (Figure 1g). To integrate the μ‐Bead EMchip with the μ‐Exo Biochip, the pattern of the coil elements was designed to correspond to the structure in the microfluidic layer. To achieve full automation of the biological assay, the motor was programmed to follow the predetermined movement commands, enabling contactless manipulation of beads and droplets to conduct the assay procedure (Figure S12, Supporting Information).

Facilitated by the integration of the μ‐Exo Biochip and μ‐Bead EMchip, a showcase of the automated biological assay on‐chip was shown in Figure 3d and Movie S1 (Supporting Information). In our automated testing workflow, the bead and droplet operations were conducted to complete the developed iME beads‐based Exo‐Aβ42 identification assay on‐chip. Specifically, after sample loading, a cluster of beads was first magnetically rotated and mixed within the introduced sample droplet to capture exosomes with CD63 (Figure 3d, **step 1**). Then, the beads were extracted across the water‐oil interface and transferred to a wash droplet for the removal of excess substances (Figure 3d, **step 2**). Subsequently, enzymes conjugated anti‐Aβ42 antibodies were specifically labelled to the target molecule Exo‐Aβ42 (Figure 3d, **step 3**). The next steps in the sequence involved three washings to minimize background interference, followed by the delivery of the labelled molecules to the detection chamber, which contained the substrate reagent to initiate the reaction (Figure 3d, **step 4**). Following the redox reaction, a stop buffer droplet was transferred to the detection chamber, where it merged and mixed with the reactive droplet to stop the reaction and amplify color development, thereby enhancing the signal (Figure 3d, **step 5**). To prevent light scattering of the magnetic beads from interfering with the sensing accuracy,^[^ ^52^ ^]^ the beads in the detection chamber were extracted. The colorimetric readout was then quantified and displayed in a sample‐to‐answer manner (Figure 3d, **step 6**).

An optical sensor model was integrated directly below the detection chamber to measure light absorbance through the signal readout droplet. The detection accuracy of the optical sensor was optimized to achieve a linear regression of *R* ^2^ = 0.995 compared to a standard microplate reader (Figure S13, Supporting Information). To validate the on‐chip assay detection and automation performance of real samples, C57BL/6J and 5×FAD transgenic mice were utilized for blood sampling and on‐chip testing (Figure 3e). The 5xFAD transgenic mouse model, which exhibits early onset and aggressive age‐dependent progression of Aβ aggregation in the brain, is one of the most widely utilized mouse models for AD research.^[^ ^67^ ^]^ Immunohistochemical staining of Aβ42 aggregates in the hippocampus of 5xFAD mice was performed to confirm the viability and reliability of the transgenic model (Figure 3f; Figure S14, Supporting Information). The immunoreactivity results from all tested 5xFAD mice brains showed robust Aβ42 deposits compared to the C57BL/6J control of the same age. Plasma samples (*n* = 20) were collected and tested on‐chip, revealing a significant difference in Exo‐Aβ42 levels between the 5xFAD group and C57BL/6J control (*p* = 0.0014, Mann–Whitney test) (Figure 3g,h). Additionally, we investigated the total Aβ42 protein levels in plasma samples (*n* = 20) using ELISA (Figure S15, Supporting Information). The results showed that the mean value of total Aβ42 protein level was higher in 5xFAD mice compared to controls. However, this difference was not statistically significant. Receiver operating characteristic (ROC) curve analysis of Exo‐Aβ42 levels in plasma measured by MEMOs and Aβ42 levels in plasma tested by ELISA (Figure S16, Supporting Information) indicated the diagnostic potential of MEMOs targeting plasma Exo‐Aβ42 biomarker for AD detection, further demonstrating the feasibility of automated capabilities of MEMOs detection.

### 2.3 MEMOs Clinical Samples Testing

**Figure 4:**
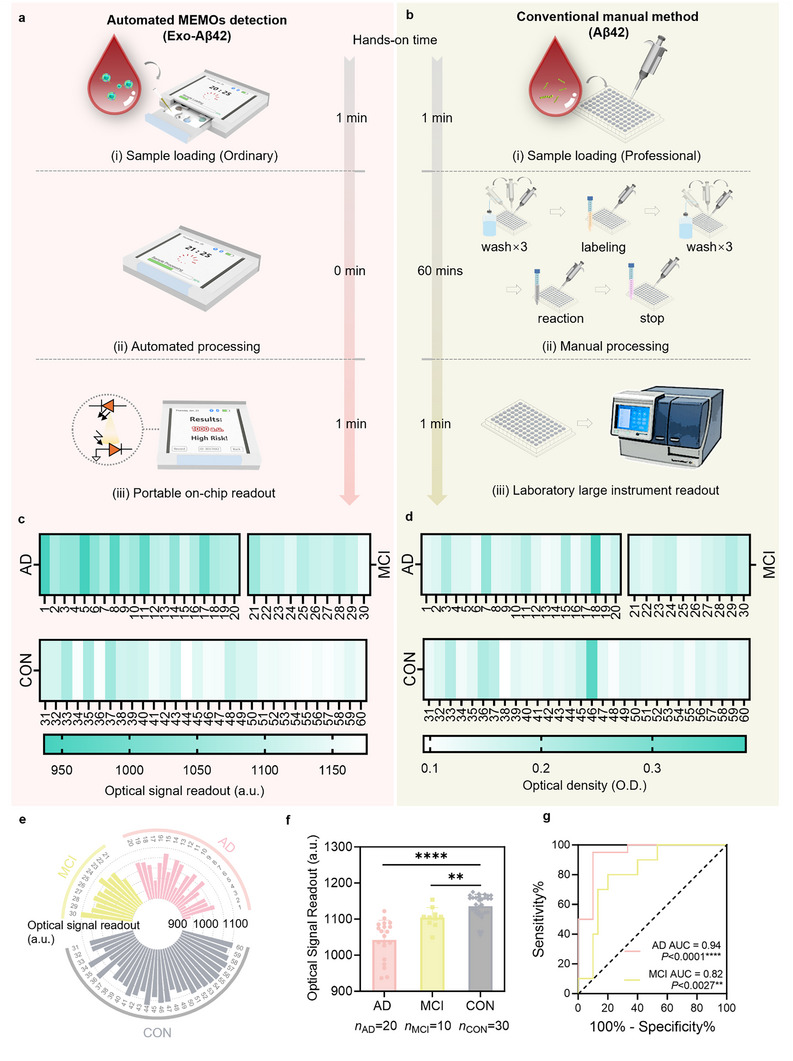
Validation of the clinical feasibility of MEMOs. a) The demonstration of the automated MEMOs detection workflow. b) The demonstration of the conventional manual method workflow.^[^ ^68^ ^]^ c) The corresponding distribution of response signals for 60 clinical samples, highlighting the changing trends associated with different stages of AD (*n* = 20), MCI (*n* = 10), and CON (*n* = 30), as tested by the automated MEMOs detection system (AD, Alzheimer's disease patients; MCI, mild cognitive impairment patients; CON, healthy controls). d) The corresponding distribution of the response signals for the same 60 clinical samples, illustrating the changing trends at various stages of AD (*n* = 20), MCI (*n* = 10), and CON (*n* = 30), as tested by the conventional manual method. e) Circle plots displaying the MEMOs sensing signal values of 60 clinical samples. f) Statistical significance analysis of the MEMOs sensing signals in AD, MCI, and CON clinical plasma samples. Statistical analysis was done using unpaired Mann–Whitney nonparametric test. *P < 0.05, **P < 0.01, ***P < 0.00, ****P < 0.0001. g) The receiver operating characteristic (ROC) curve analysis of 60 clinical samples based on MEMOs tested plasma Exo‐Aβ42 levels.

To enhance the accessibility of MEMOs in various settings, including hospitals, clinics, and even at home, a user‐friendly workflow was adopted, consisting of the following three simple steps (**Figure** **4**a): i) Load the collected microliter‐volume sample into the μ‐Exo Biochip; ii) Insert the μ‐Exo Biochip to initiate the automated sample processing and detection procedure; iii) View the readout signals and interpreted results on the screen. Compared to the conventional laboratory‐based methods (Figure 4b), MEMOs provided a user‐friendly workflow that did not require specialized training for sample handling. Additionally, MEMOs, with its automated sample processing capability, reduced processing hands‐on time from the conventional 60 min^[^ ^68^ ^]^ to 0 min, completely eliminating the need for manual pipetting during washing, labeling, reaction, and stopping steps. Moreover, MEMOs offered on‐chip readout, overcoming the limitations associated with bulky laboratory signal readers. In addition to its easy operation workflow and minimized hands‐on time, the overall manufacturing price of MEMOs, including constituent hardware and consumables, is cost‐effective (Note S2 and Table S1, Supporting Information). The basic cost price of each MEMOs test is ≈5 USD, which is two orders of magnitude lower than the service cost of existing Alzheimer's disease diagnostic tests on the market (Table S2, Supporting Information). Therefore, the integration of the μ‐Exo Biochip and μ‐Bead EMchip enabled MEMOs to substantially simplify manual operation steps, eliminate complex hands‐on processing time, and reduce manufacturing cost, improving its diagnostic accessibility.

The performance of MEMOs for clinical testing feasibility was investigated using a total of 60 clinical samples, consisting of 20 Alzheimer's samples, 10 mild cognitive impairment samples, and 30 healthy control samples. The CD63 levels of clinical samples in each group were analyzed by ELISA, and no significant differences were found among the AD, MCI and CON samples (Figure S17, Supporting Information), indicating that the Exo‐Aβ42 biomarkers were assessed under the assumption that exosomal CD63 levels did not vary among clinical samples. To evaluate the clinical performance of MEMOs, each plasma specimen was loaded into MEMOs for Exo‐Aβ42 detection and simultaneously tested on a microplate for plasma total Aβ42 detection using a commercial ELISA kit for comparison. The sensing signals of Exo‐Aβ42 protein detected on‐chip for the 60 samples were presented in Figure 4c,e, while the plasma total Aβ42 detection results were shown in Figure 4d and Figure S18a (Supporting Information). In the MEMOs Exo‐Aβ42 group, it was observed that AD samples exhibited significantly lower readout signals compared to the healthy control group (*P* < 0.0001, Mann–Whitney test), and the differences between MCI and CON were also notable (*P* < 0.0027, Mann–Whitney test) (Figure 4f). However, the plasma total Aβ levels did not show any significant differences among the three groups (Figure S18b,c, Supporting Information). These results indicated a progressively rising trend in plasma Exo‐Aβ42 levels across the CON, MCI, and AD stages of AD, suggesting the potential of plasma Exo‐Aβ42 as a predictor for differentiating multiple stages of AD progression.

ROC analysis was also conducted to evaluate the diagnostic potential of our platform for AD and to determine the critical values of Exo‐Aβ42 protein marker among the AD, MCI, and CON groups (Figure 4g). The area under the curve (AUC) value was found to be 0.94, with a *P* value of <0.0001 for the comparison between AD and CON groups, indicating excellent diagnostic accuracy (AUC ≥ 0.9) for AD, based on the established guidelines for AUC interpretation.^[^ ^69^ ^]^ Additionally, the AUC value was 0.82, with a *P* value of <0.01 for the MCI vs CON groups, which was interpreted as demonstrating good and considerable testing performance, further suggesting the capability of our platform for monitoring AD progression. The ROC curve analysis indicated that our platform can diagnose AD patient groups with a sensitivity of 95% and specificity of 90% (cut‐off value ≤ 1104) and can distinguish MCI patient groups from healthy CON groups with a sensitivity of 80% and specificity of 80% (cut‐off value ≤ 1115). These results unraveled the potential of MEMOs to automate AD blood testing and suggested that it provided an accessible and convenient pathway toward diagnosing and monitoring the progression of the disease.

## 3 Conclusion

This work presents a fully integrated and automated system, MEMOs, for detecting the AD blood biomarker, Exo‐Aβ42 protein. MEMOs automatically handles biomarker processing, analysis, and data readout using a programmed actuation system, thereby eliminating the necessity for manual intervention. In contrast to previous technologies, which typically function within traditional laboratory settings, MEMOs provides a more accessible alternative suitable for a wider range of settings. This enables non‐specialists to conduct home testing while still preserving high sensitivity and specificity in diagnosing Alzheimer's disease. The benefits of MEMOs stem from a comprehensive combination of the following components: 1) a μ‐Exo Biochip, encapsulated within a magnetic field‐responsive assay, is specially designed to target dual marker sites of the Exo‐Aβ42 protein, achieving detection limits as low as several picograms per milliliter; 2) a μ‐Bead EMchip, featuring a programmable navigation matrix, is engineered for robustly addressable magnetic motor actuation in accordance with predetermined commands; and 3) the integrated system facilitates contactless control of both bead and droplet movements, enabling diverse functionalities and the execution of immunosensing operations in a fully automated manner.

Utilizing MEMOs, we assess the levels of blood Exo‐Aβ42 in both animal models (including healthy and AD transgenic mice) and clinical samples (plasma from healthy individuals, patients with mild cognitive impairment, and Alzheimer's patients). Our findings reveal significant correlations between blood Exo‐Aβ42 levels and the progressive stages of AD, underscoring the potential of MEMOs as an accessible point‐of‐care diagnostic device for monitoring the development of AD. MEMOs features 1) cost‐effective manufacturing, lowering the expense of each MEMOs test to ≈5 USD; 2) minimized hands‐on time, eliminating the entire manual operation to 2 min; and 3) a straightforward operational workflow, requiring only 3 simple operation steps. These three features facilitate MEMOs as an efficient tool for evaluating blood Exo‐Aβ42 levels. Despite the pressing need for blood testing for AD, such assessment has not been widely implemented in routine clinical practice, primarily due to a lack of established protocols rather than a lack of need.^[^ ^23^, ^25^, ^26^, ^28^, ^70^ ^]^ Recent advances in the exploration of AD blood biomarkers, such as the newly validated markers p‐tau^217^ and p‐tau^181^,^[^ ^71^, ^72^ ^]^ underscore this urgent need. These developments also reflect transformative advances in the management of AD. To promote wide implementation of AD blood tests at large scales, it is necessary to develop fully automated, commercially available, and easily accessible platforms. MEMOs can meet this need by providing clinical insights during early medical assessments to alleviate the pressure on healthcare systems and facilitate the rational allocation of medical resources. Furthermore, the results obtained from MEMOs can be interpreted in conjunction with other diagnostic modalities, such as cerebrospinal fluid analysis and positron emission tomography, to enhance clinical decision‐making.

On a broader level, the adaptability of MEMOs can be improved to support large‐scale, high‐throughput, and multiplexed biomarker detection. From a system perspective, the current configuration is designed to execute an iME beads‐based assay for Exo‐Aβ42 identification. The patterns in the μ‐Exo Biochip and the navigation matrix in the μ‐Bead EMchip can be easily modified to adapt to various test procedures. Additionally, the integrated readout module currently relies on a colorimetric measurement sensor. Future enhancements could include the automation and integration of other optical and electrochemical signal readout models to meet diverse signal generation requirements. Through reconfigurable assay actuation patterns and elaborate signal acquisition approaches, MEMOs can theoretically provide multiple parallel detection channels, hence enabling large‐scale high‐throughput screening. From an application perspective, MEMOs can be customized for on‐demand analysis of other disease‐associated exosome and/or protein biomarkers. Furthermore, leveraging the technological capabilities for large‐scale and high‐throughput actuation, MEMOs holds the potential for simultaneous analysis of multiple targets, thereby enhancing healthcare efficiency. The potential incorporation of artificial intelligence algorithms can further streamline the operational steps required for multiplexed testing, facilitating more intricate bioanalytical applications.

## 4 Experimental Section

### Materials

Plasma sample from healthy individuals (*n* = 30) (IPLASK2E2ML, Innovative Research), Alzheimer's disease patients (*n* = 20) (IPLASALZ, Innovative Research; HUMANPLK2, BioVIT; B2012893P, Molecular Depot), and patients with mild cognitive impairment (*n* = 10) (B2014101M, Molecular Depot), were obtained from Innovative Research, Inc. (Novi, MI, USA), BioIVT LLC. (Westbury, NY, USA) and Molecular Depot LLC (San Diego, USA) to minimize the bias in the plasma sample treatment process. Mild cognitive impairment represents an early stage of memory loss, characterized by less severe symptoms compared to Alzheimer's disease, which was confirmed through Mini‐Mental State Examination (MMSE) screening. The confirmation of AD is based on clinical diagnosis. All human plasma samples were stored at −20 °C. Human primary anti‐CD63 antibody (MA5‐30187), APC goat anti‐Rabbit IgG (H+L) secondary antibody (A‐10931), and 10 nm AuNPs conjugated goat anti‐Rabbit IgG (H+L) secondary antibody (A‐31566), were obtained from ThermoFisher Scientific. Mouse primary anti‐CD63 antibody (ab281516), mouse anti‐beta Amyloid 1‐42 antibody (ab280993), and human anti‐beta Amyloid 1‐42 antibody (ab240233) were obtained from Abcam limited. Total Exosome Isolation Reagent (from cell culture media) (4478359) was obtained from ThermoFisher Scientific. Exosome FITC staining (EXOFLOW800A‐1) and the FluoroCet Exosome Quantitation Kit (FCET96A‐1) were obtained from System Biosciences.

### Cell Culture

Human neuroblastoma cell line SH‐SY5Y (RRID: CVCL_0019) was obtained from the American Type Culture Collection (ATCC, CRL‐2266). SH‐SY5Y cell line was tested negative for mycoplasma contamination. SH‐SY5Y cells, at passages 1‐15, were cultured in a medium consisting of Minimum Essential Medium (MEM, Gibco) and Ham's F‐12 Nutrient Mix with GlutaMAX Supplement (F‐12, Gibco) in a 1:1 ratio. The culture medium was supplemented with 10 % exosome‐depleted fetal bovine serum (EXO‐FBS‐250A‐1, System Biosciences) and 1 % penicillin–streptomycin (15070063, Gibco). The cells were maintained at 37 °C in a humidified atmosphere containing 5 % CO_2_.

### Exosome Isolation

SH‐SY5Y cells at passages 1‐15 were cultured in exosome‐depleted medium with 10% exosome‐depleted FBS (EXO‐FBS‐250A‐1, System Biosciences) for 48 h. The cell culture media was harvested and filtered through a 0.2‐µm membrane filter. The filtered media was then centrifuged at 2000 ×g for 30 min to remove cell debris. The supernatant was transferred to a new tube, mixed with total exosome isolation reagent (4478359, Invitrogen) according to the manufacturer's protocol, and incubated at 4 °C overnight. Following incubation, the sample was centrifuged at 10000 ×g for 1 h at 4 °C. The supernatant was discarded, and the exosomes were collected in the pellet at the bottom of the tube. PBS was used to resuspend the exosome pellet for subsequent experiments.

### Preparation of iME Beads‐Based Exosome Identification Assay

A theoretical estimation of the amount of primary antibody, biotin, and magnetic beads per test was provided in Note S3 (Supporting Information), where the binding site of the magnetic beads, the steric effect of the IgG antibody, and the target molecule concentration in the sample were considered. The experimental functionalization was guided by the theoretical estimation to strike the balance between maximizing effective binding and minimizing non‐specific binding. The anti‐CD63 primary antibody was first conjugated with biotin using a biotinylation kit (ab201796, Abcam) following the kit protocol and our theoretical estimation. Based on the guidance of estimation, we identified the optimal functionalization conditions, which involved incubating the biotin‐conjugated CD63 primary antibody (0.07 mg ml^−1^) with streptavidin‐coated magnetic beads (4 mg ml^−1^, 11206D, Invitrogen) in 100 µl of PBS for 2 h at room temperature under constant rotation and protected from light. Then, the beads were washed three times with PBS containing 0.4 % Block Ace (Bio‐Rad), and blocked with PBST buffer containing 4 % Block Ace for 1 h at room temperature to reduce background noise signal and prevent non‐specific binding. After blocking, the coated beads were concentrated to 10 mg ml^−1^ in buffer A SignalPlus Antibody Enhancer (GeneTex) to further enhance the antibody‐antigen binding affinity. The iME beads were then ready to use for exosome capture. To generate a signal, Horseradish peroxidase (HRP) was conjugated to the anti‐Aβ42 antibody using a HRP antibody conjugation kit (ab102890, Abcam) according to the manufacturer's protocol. The HRP conjugated anti‐Aβ42 antibody was diluted 1:1000 in buffer B SignalPlus Antibody Enhancer (GeneTex) before use to improve the target sensitivity. Wash buffer was prepared using PBST containing 0.4 % Block Ace to keep the background signal at a relatively low‐level and not affect the assay performance. TMB buffer (34028, ThermoFisher Scientific) and stop buffer (SS03, ThermoFisher Scientific) were used to amplify the optical signal and stop the reaction. Non‐coated polystyrene magnetic microbeads (49664, Sigma–Aldrich) were used to adjust the bead loading mass, facilitating the operation of beads and droplets.

### Fabrication of the Microfluidic Exosome‐Detection Biological Chip (μ‐Exo Biochip)

The μ‐Exo Biochip was fabricated by laser engraving different patterns on two polytetrafluoroethylene (PTFE) substrates, which were then sandwiched between two transparent polyethylene terephthalate (PET) film sheets. This configuration encapsulated mineral oil (5904, Sigma–Aldrich) and assay reagents within a sealed environment, including one 8 µl beads droplet, four 50 µl wash droplets, one 50 µl droplet of HRP‐conjugated anti‐Aβ42 antibody, one 50 µl TMB droplet, and one 50 µl stop buffer droplet with beads for droplet transportation. The substrates were assembled by using double‐sided tape to construct chamber heights of ≈3 mm.

### Design of the Microbead Operation Electromagnetic Chip (μ‐Bead EMchip)

The μ‐Bead EMchip was designed based on a four‐layer PCB^[^ ^66^ ^]^ and consisted of 165 active coil elements. Each element featured a four‐turn coil (2 mm × 2 mm) that was stacked on the top three layers of the PCB, with a 0.1‐mm gap separating it from adjacent elements. Each coil element can be powered independently by a direct current (DC) of 0.4 A, generating a localized magnetic force to attract the magnetic motor. A total of 21 programmable switch ICs (MAX14662, Maxim Integrated) were mounted on the bottom layer of the PCB, with each output port connected to individual coil element to selectively activate the coil elements. The switch ICs were connected in a serial peripheral interface Daisy‐Chain configuration to Arduino Nano. A simple path‐finding algorithm was embedded in the Microcontroller. Target destinations or operations sent from the user interface were processed by the path‐finding algorithm, then translated into serial peripheral interface commands by the Arduino and subsequently transmitted to the switch ICs for automated addressable activation of the coil elements.

A blue LED (L450R‐04, USHIO) and an optical sensor (3.3 V, BH1721, ROHM Semiconductor) were used to measure the optical signal. The optical sensor was connected to Arduino Nano through Inter‐Integrated Circuit protocol (I2C). A 2 mm × 2 mm board cutout was centrally positioned within the detection region of the μ‐Bead EMchip. The light emitted by the LED passed through the droplet and then reached the optical sensor through the cutout. To minimize the baseline signal drift caused by ambient light variations and component warm‐up effects, the readout was calibrated. During MEMOs initialization, a 10‐second stabilization period was implemented where the ambient light intensity was sampled 100 times at 10 Hz through the optical pathway. The acquired signals were subjected to digital filtering to attenuate high‐frequency noise components, followed by computation of the arithmetic mean that established the reference baseline intensity (*I_ref_*). After sample processing, prior to signal readout, the established protocol was reapplied to capture instantaneous background intensity (*I_bg_*). Then, the optical signal intensity of the tested sample (*I_raw_*) was measured and filtered continuously over a duration of 10 s to ensure measurement accuracy. To enable dynamic compensation of baseline drift, the final readout signal (*I_readout_*) was derived through the normalized calibration relationship:

(1) $$I_{readout}=I_{raw}\frac{I_{ref}}{I_{bg}}$$

### Preparation of Synthesized Exosome‐Amyloid Beta Associations

Lyophilized Aβ42 protein (rPeptide) was resuspended in PBS buffer, containing the exosome‐isolated pellet, and rotated at 4 °C for 20 h to facilitate the formation of associations. After incubation, the initial buffer was diluted with PBS to achieve the desired concentration and was used immediately. Aliquots of 100 µl were used for on‐chip testing.

### Flow Cytometry Analysis

To characterize the immune coating efficiency of magnetic beads, an APC‐conjugated secondary antibody was used to label the primary antibody. Briefly, primary antibody‐coated beads (1 mg ml^−1^ PBS) were incubated with secondary antibody (10 µg ml^−1^) for 1 h at room temperature under constant rotation and protected from light. Blank non‐coated beads were used as controls for the non‐specific binding test. Then, the beads were washed three times in PBS containing 0.4 % Block Ace, after which the pellets were resuspended in PBS for flow cytometry analysis using a BD FACSAria III Cell Sorter. To assess the exosome capture performance of the immune beads, a commercial exosome purification kit was used. Briefly, the exosome‐captured beads (100 µg) were resuspended in 250 µl of exosome staining buffer containing 10 µl FITC exosome stain and placed on ice for 3 h. The tube was flicked every 30 min to ensure gentle mixing. Blank primary antibody‐coated beads were used as controls for the non‐specific FITC exosome dye binding test. The beads were then washed three times in PBS containing 0.4 % Block Ace, after which the pellets were resuspended in PBS for flow cytometry (BD FACSAria III Cell Sorter). Analyses were performed using FlowJo V10 Software, with single beads gated based on their forward and side scattering properties.

### Electron Microscopy

The energy dispersive X‐ray spectroscopy images of the AuNPs conjugated secondary antibody labelled iME beads were obtained using a FEG Scanning Electron Microscope (Hitachi S‐4800) without Au sputtering on the samples. Exo‐Aβ42 samples were fixed with 4 % paraformaldehyde (I28800, ThermoFisher Scientific), washed three times, and transferred onto a nickel grid. After that Aβ42 proteins were subjected to double immune‐labelling using Aβ42 primary antibody (ab180956, Abcam) and 10 nm AuNPs conjugated secondary antibody (A‐31566, ThermoFisher Scientific). The labeled samples were washed and contrast stained. Dried samples were imaged with a Scanning Transmission Electron Microscope (FEI Tecnai G2 20). Exosomes and Aβ42 aggregates were fixed with 4 % paraformaldehyde and transferred onto a copper grid, washed and contrast stained. Dried samples were imaged with a transmission electron microscope (Philips CM100).

### Confocal Microscopy

The fluorescence images of the fluorescent beads were obtained using a confocal and multiphoton microscope (Nikon NEW AX R high speed confocal, Nikon Ti2‐E motorized inverted microscope) by depositing the beads onto a 0.17 mm confocal glass slide. The fluorescence images were recorded by collecting light in the 500–600 nm under excitation by a 488 nm laser.

### Animal and Human Plasma Sample Analysis

C57BL/6J mice and 5xFAD mice aged between 7 and 9 months were housed in the Centre for Comparative Medicine Research (CCMR) at the University of Hong Kong, which is accredited by the Association for Assessment and Accreditation of Laboratory Animal Care International. The use of animals was approved by the Department of Health, Hong Kong, and the Committee on the Use of Live Animals in Teaching and Research (CULATR) at the University of Hong Kong (#12‐127). Mice were housed in a temperature‐controlled room between 20 and 22 °C, with humidity of 50 ± 10 % under a 12/12 h light/dark cycle. Food and water were provided ad libitum, and all possible efforts were made to minimize the number of animals being used and animal suffering. 0.6 ml of blood was collected via cardiac puncture into Eppendorf tubes precoated with 0.5 M EDTA. Blood was then centrifuged at 1200 × g for 15 min at 4 °C. Plasma was collected carefully and stored at −80 °C until use. 100 µl aliquots of mouse plasma were thawed to room temperature and then loaded onto the chip for testing. Plasma samples from healthy individuals, Alzheimer's disease patients, and mild cognitive impairment patients were thawed to room temperature before use. 100 µl aliquots of human plasma were loaded onto the chip for testing.

### Statistical Analysis

All statistical analyses were performed using GraphPad Prism software version 10.0. Animal samples (*n* = 20) from 5×FAD transgenic mice and C57BL/6J mice, along with clinical samples (*n* = 60) were analyzed using unpaired Mann–Whitney nonparametric test. *p* < 0.05 was considered statistically significant for all analyses.

## Conflict of Interest

The authors declare the following competing interests: H.C. Shum is a scientific advisor of EN Technology Limited, MicroDiagnostics Limited, PharmaEase Tech Limited, and Upgrade Biopolymers Limited in which he owns some equity, and also a founding centre director and co‐director of the research centre, namely Advanced Biomedical Instrumentation Centre Limited. The works in the paper are however not directly related to the works of these entities. The authors declare no other competing financial interests.

## Supporting information



Supporting Information

Supplemental Movie 1

## Data Availability

The data that support the findings of this study are available in the supplementary material of this article.
