# Supplementary material for: An Automated Micro‐Immunobeads‐Based Electromagnetic Operation System (MEMOs) for Blood Testing of Alzheimer's Disease
Source: Adv Sci (Weinh). 2025 Aug 18;12(41):e09376. doi: 10.1002/advs.202509376 (PMC12591217; doi:10.1002/advs.202509376)
Supplement: Supplementary file 1 — Supporting Information [file ADVS-12-e09376-s001.docx]

Supplementary Information

**An automated micro-immunobeads-based electromagnetic operation system (MEMOs) for blood testing of Alzheimer’s disease**

*Xiaoxue Fan, Xinyu Ye, Jiangrui Xu, Tianjiao Mao, Ruotong Zhang, Xinyuan Zhou, Christina C. K. Au Yeung, Chang Li, Raymond Chuen-Chung Chang^*^, Haisong Lin^*^, Ho Cheung Shum^*^*

This file includes:

**Note S1 |** Force balance model of the bead and droplet operation zones.

**Note S2 |** The comparison between MEMOs and ELISA.

**Note S3 |** Theoretical functionalization process of immune beads.

**Figure S1 |** Development and evaluation of iME beads.

**Figure S2 |** Scanning electron microscope (SEM) images of blank beads and iME beads with AuNPs conjugated antibody dye.

**Figure S3 |** SEM-EDS analysis elemental mapping of blank beads and blank beads with AuNPs conjugated antibody dye.

**Figure S4 |** Characterization of extracellular vesicles derived by SH-SY5Y neuronal cells.

**Figure S5 |** Flow cytometer characterization of exosome capture performance of iME beads.

**Figure S6 |** Fluorescent microscope images of iME beads with FITC exosome dye and iME beads captured with FITC labelled exosomes.

**Figure S7 |** Characterization of the prepared Exo-Aβ42 proteins.

**Figure S8 |** Exosome quantification standard curve.

**Figure S9 |** Optimization of iME beads-based Exo-Aβ42 identification assay on-chip.

**Figure S10 |** The corresponding calibration process of MEMOs.

**Figure S11 |** Contact angles of different substrates.

**Figure S12 |** Administration and implementation of μ-Bead EMchip manipulation at software and hardware levels.

**Figure S13 |** Characterization of optical sensor readout versus standard microplate reader readout.

**Figure S14 |** Immunohistochemical staining of Aβ42 aggregates in the hippocampus of 5xFAD and C57BL/6J mice.

**Figure S15** | Commercial ELISA kit characterization of plasma total Aβ42 protein levels in animal samples.

**Figure S16 |** Receiver operating characteristic (ROC) curve analysis of Exo-Aβ42 levels in plasma measured by MEMOs and Aβ42 levels in plasma tested by ELISA in animal samples.

**Figure S17 |** Characterization of the CD63 protein levels in exosome of human samples.

**Figure S18 |** Characterization of plasma total Aβ42 protein levels in human samples.

**Table S1 |** The list and quantity of components and estimated cost of a MEMOs.

**Table S2 |** Service cost comparison between commercialized blood tests and conventional tests in Alzheimer's disease diagnosis.

**Movie S1 |** Automated MEMOs testing.

**Supplementary Note 1 | Force balance model of the bead and droplet operation zones**^[1–3]^*

A. Take both the droplet and the bead cluster as an object:

The external forces are simplified as: the magnetic force $F_{m}$acts as the driving force, and the friction force $F_{f}$ acts as the resistive force.


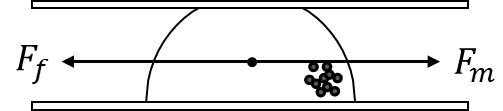

$$F_{m}={K_{m}V_{p}\Delta_{\chi}\cong K}_{m} \left( \frac{M}{\rho} \right)\chi_{p}$$

where $K_{m}$ is related to the properties of the applied magnetic field^[4]^, $V_{p}$ is the volume of the particle and $\Delta_{\chi}$ is the magnetic susceptibilitiy differences between particle and surrondings. The volume of the particle $(V$) is related to the mass of the particles $(M$) and the density of the particles $(\rho$). The magnetic susceptibility of the surrounding buffer in our system is negligible, thus here we make the assumption that the magnetic susceptibilitiy differences${(\Delta}_{\chi})$ equals to the magnetic susceptibility of the particles ($\chi_{p}$).

$$F_{f}\cong K_{f}R_{d} \mu_{oil} V$$

where $K_{f}$ is a friction constant^[2,5]^, $R_{d}$ is the radius of the contacted curvature between the droplet and substrate, $\mu_{oil}$ is the viscosity of the oil and $V$ is the velocity of the droplet.

B. Take the droplet or the bead cluster as an object:

The internal force $F_{inter}$ should be taken into consideration. $F_{inter}$ is generated due to the interaction between the bead cluster and the droplet causing the deformation of the droplet surface, which is related to the interfacial tension^[6,7]^. The greater deformation results in larger internal force. The internal force $F_{inter}$ is defined to act on the bead cluster, and $F_{inter}^{'}$, as the interaction force of $F_{inter}$, is defined to act on the droplet. When bead cluster is extracted from the droplet, the internal force reaches its maximum $F_{inter, max}$.

$$F_{inter}= F_{inter}^{'}= L\cdot\gamma_{d-o}$$

$$F_{inter, max}= F_{inter, max}^{'}=(6^{\frac{1}{3}}\pi^{\frac{2}{3}}) {(\frac{M}{\rho})}^{\frac{1}{3}}\cdot\gamma_{d-o}$$

where $L$ is the length of the deformed curvature and $\gamma_{d-o}$ is the interfacial tension between droplet and oil. When the internal force reaches its maxmium, the deformed curvature length $(L)$ is estimated to equal to the circumference of the bead cluster sphere.

1. Take bead cluster as an object:


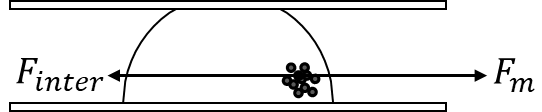


1. Take droplet as an object:


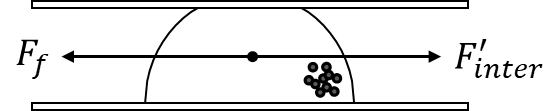


C. Conditions of three different actuation zones

1. At the no engagement zone, where neither beads nor droplet is actuated:

Take bead cluster as an object: $F_{m}{=F}_{inter}, F_{inter}< F_{inter, max}$

Take droplet as an object: $F_{inter}\leq F_{f}$

1. At the bead operation zone, where beads are extracted from droplet, while the droplet stays stationary:

Take bead cluster as an object: $F_{m}> F_{inter}, F_{inter}= F_{inter, max}$

Take droplet as an object: $F_{inter} \leq F_{f}$

1. At the droplet operation zone, where both beads and droplet are moved steadily:

Take bead cluster as an object: $F_{m}= F_{inter}, F_{inter}< F_{inter, max}$

Take droplet as an object: $F_{inter} = F_{f}$

*Only horizontal components of the forces are listed.

**Supplementary Note 2 | The comparison between MEMOs and ELISA**

Compared with ELISA technology, MEMOs has its unique advantages. The integration of the μ-Exo Biochip and μ-Bead EMchip enables MEMOs to substantially streamline manual operations, eliminate complex hands-on processing time, and reduce manufacturing cost, thereby improving its diagnostic accessibility. Through the systematic optimization of the compatibility between the automation-compatible assay and the actuation system, MEMOs can attain detection sensitivity at the level of pg ml^-1^, which is comparable to typical detection values of commercial ELISA kits. However, the conventional hands-on ELISA is usually conducted in a laboratory setting due to the requirement for bulky instruments^[8]^. Skilled technicians are needed to spend considerable time on each of the processing steps, including washing, labelling, stopping, etc. In contrast, MEMOs provides automated procedures that reduce result turnaround time, improve public accessibility, and minimize errors associated with manual operations. Robotic ELISA workstation is developed to eliminate the manual operation time of conventional hands-on ELISA by automating the pipetting and transferring of different reagents^[9]^. Nonetheless, the robotic ELISA workstations are bulky and require large spaces. Despite the availability of compact versions, these workstations are still generally utilized within laboratories. Conversely, MEMOs is portable and can be employed in different settings. Quanterix Simoa, an advanced and highly sensitive ELISA workstation, has been launched. The principle of Quanterix Simoa is based on beads-based digital ELISA. Following the implementation of a robotic system to execute standardized beads-based assay protocols, single beads are isolated into small reaction chambers, and digital imaging is subsequently utilized to quantify the signal. Beads-based digital ELISA leverages physical confinement enabled single-molecular resolution and Poisson distribution to analyse the random distribution of single bead signal, thereby achieving ultra-sensitivity at the sub-femto level^[10]^, around thousands of times more sensitive than conventional hands-on ELISA, robotic ELISA, and MEMOs. However, in comparison, MEMOs is cost-effective with approximately $5 per test (Table S1) and the results are available in time (Table S2), which broaden its public accessibility.

**Supplementary Note 3 | Theoretical functionalization process of immune beads**

1. Determine the volume of antibody used per test

The concentration of exosomes in the blood is approximately ~10^8^ particles ml^-1[11,12]^. In our system, a sample volume of 100 μl was selected for each test, which theoretically contains around 10^7^ exosomes. Therefore, the minimum molar amount of primary antibody *m_antibody_* used per test in our case was estimated at the level of 10^7^ N_A_^-1^ (N_A_ is the Avogadro constant). If the antibody quantity is too low, it may cause insufficient binding to the bead surface, thereby reducing capture efficiency and increasing the risk of non-specific binding^[13,14]^. Conversely, if the amount of the primary antibody is too high, it may cause antibody aggregation and denaturation, significantly compromising both stability and functionality^[15]^.

The volume of antibody $V_{antibody}$needs to be biotinylated per test:

$$V_{antibody}= \frac{m_{antibody}\cdot{MW}_{antibody}}{C_{antibody}}$$

where ${MW}_{antibody}$ is the molar weight of antibody, which is usually provided in kDa unit; $C_{antibody}$ is the mass concentration of antibody, which can be obtained from the manufacturer.

2. Determine the volume of biotin molecules used to conjugate the antibody

We choose to use streptavidin and biotin conjugation to functionalize the magnetic beads. To biotinylate the specific amount of antibody, the volume of biotin used is considered as:

$$V_{biotin}=\frac{K_{active}{\cdot MW}_{biotin}\cdot m_{antibody}}{C_{biotin}}$$

where ${MW}_{biotin}$ is the molar weight of biotin, $C_{biotin}$ is the mass concentration of biotin, which can be obtained from the manufacturer, and $K_{active}$ is the ratio of biotin to antibody. Generally, each antibody can be labelled with 3-5 biotins. During biotinylating, the ratio of biotin to antibody $K_{active}$ can be affected by the antibody concentration^[16]^. Empirically, $K_{active}$ is 20 when the mass concentration of IgG antibody is around 1-3 mg ml^-1^ to guarantee the labelling efficiency^[17]^. During the experiment, the buffer used must not contain sodium azide, BSA, glycine, Tris, or any other additives with free amino groups to avoid affecting the conjugation.

3. Determine the amount of functionalized beads

The volume of beads that can be functionalized in theory is considered as:

$$V_{beads}= \frac{m_{antibody}\cdot N_{A}}{K_{steric} S_{SA}C_{beads}}$$

where $K_{steric}$ is the blocking of antibody binding by steric hindrance, $S_{SA}$ is the streptavidin site of beads, which is typically provided by the manufacturer in units of particle mg^-1^, and $C_{beads}$ is the mass concentration of beads. The steric hindrance^[18,19]^ arises when large antibody groups occupy a large amount of space on the bead surface and physically hinder other antibodies from binding to their sites or even to their target antigens. In theory, one streptavidin molecule can bind to four biotin molecules^[20,21]^. However, the binding sites of streptavidin and biotin are also affected by steric hindrance. Due to this, in our case, the $K_{steric}$ is considered as 0.3.

In summary, the above theoretical estimation of the magnetic bead functionalization process provides us with guidance for optimizing the magnetic bead capture performance through experiments.


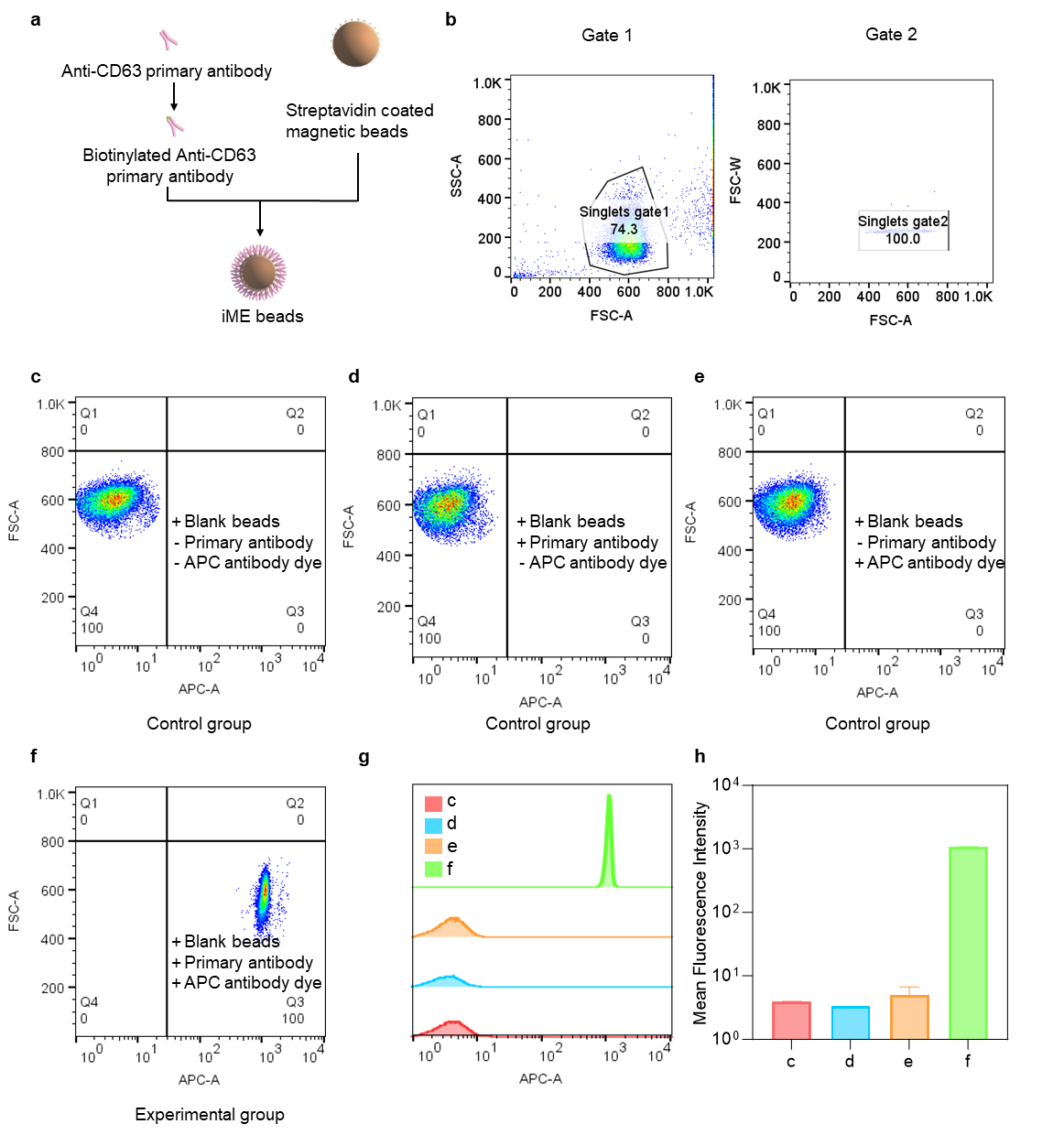


**Figure S1 | Development and evaluation of iME beads. a,** Functionalization process of the immune magnetic exosome beads (iME beads). **b,** Gating strategies for the selection of single beads. **c-h,** Flow cytometer characterization of the functionalization process of iME beads. Flow cytometry dot-plot analysis of blank beads **(c)**, iME beads **(d)**, blank beads with APC antibody dye **(e)**, and iME beads with APC antibody dye **(f)**, showing no cross-reaction between blank beads and APC antibody dye. Histogram **(g)** and mean **(h)** of the APC signal intensities of c, d, e, and f (*n* = 3)**.**


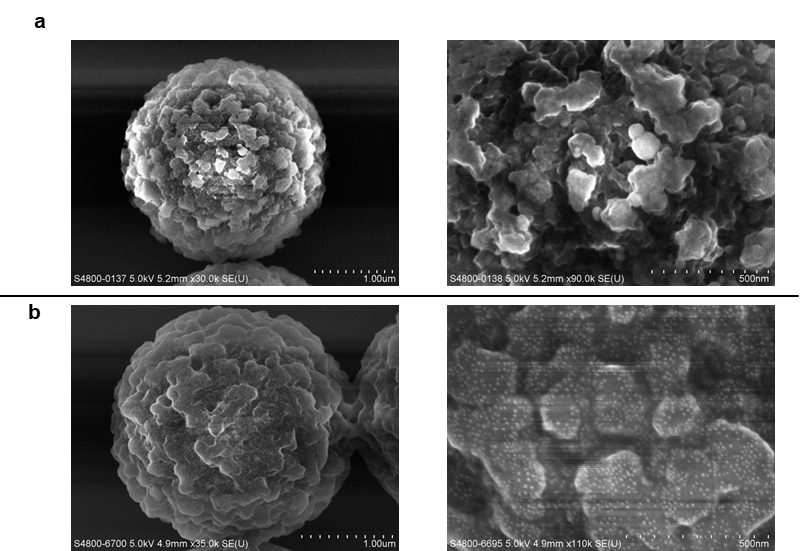


**Figure S2 | Scanning electron microscope (SEM) images of blank beads (a) and iME beads with AuNPs conjugated antibody dye (b).**


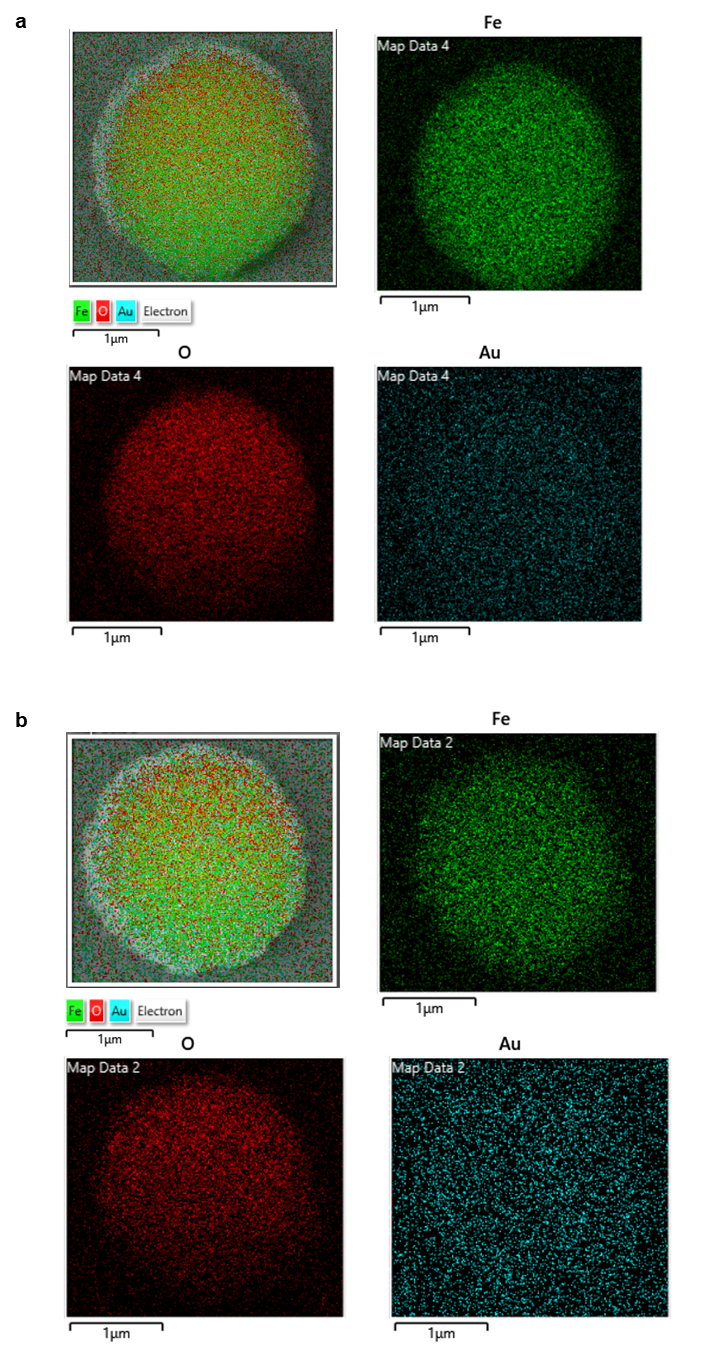


**Figure S3 | SEM-EDS analysis elemental mapping (Fe - green, O - red, Au - blue) of blank beads (a) and blank beads with AuNPs conjugated antibody dye (b).** The results indicate no cross-reaction between blank beads and AuNPs dye.


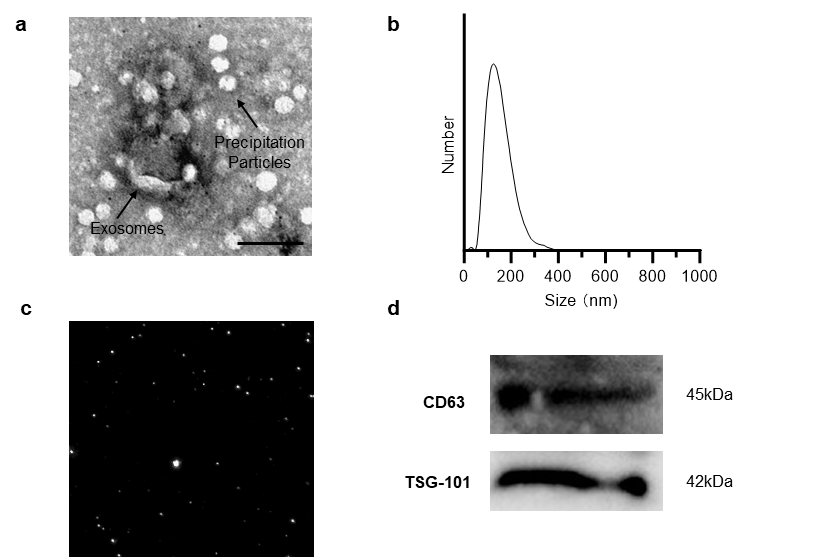


**Figure S4 | Characterization of extracellular vesicles derived by SH-SY5Y neuronal cells. a,** Transmission electron microscope of extracellular vesicles precipitated from SH-SH5Y cells. Scale bar: 100 nm. **b,** Size distribution of the isolated extracellular vesicles determined by nanoparticle tracking analysis, showing a mean value around 150 nm. **c**, Optical image of the light scattering extracellular vesicles determined by nanoparticle tracking analysis. **d,** Western blotting analysis of the isolated extracellular vesicles for exosomal markers (CD63 and TSG-101).


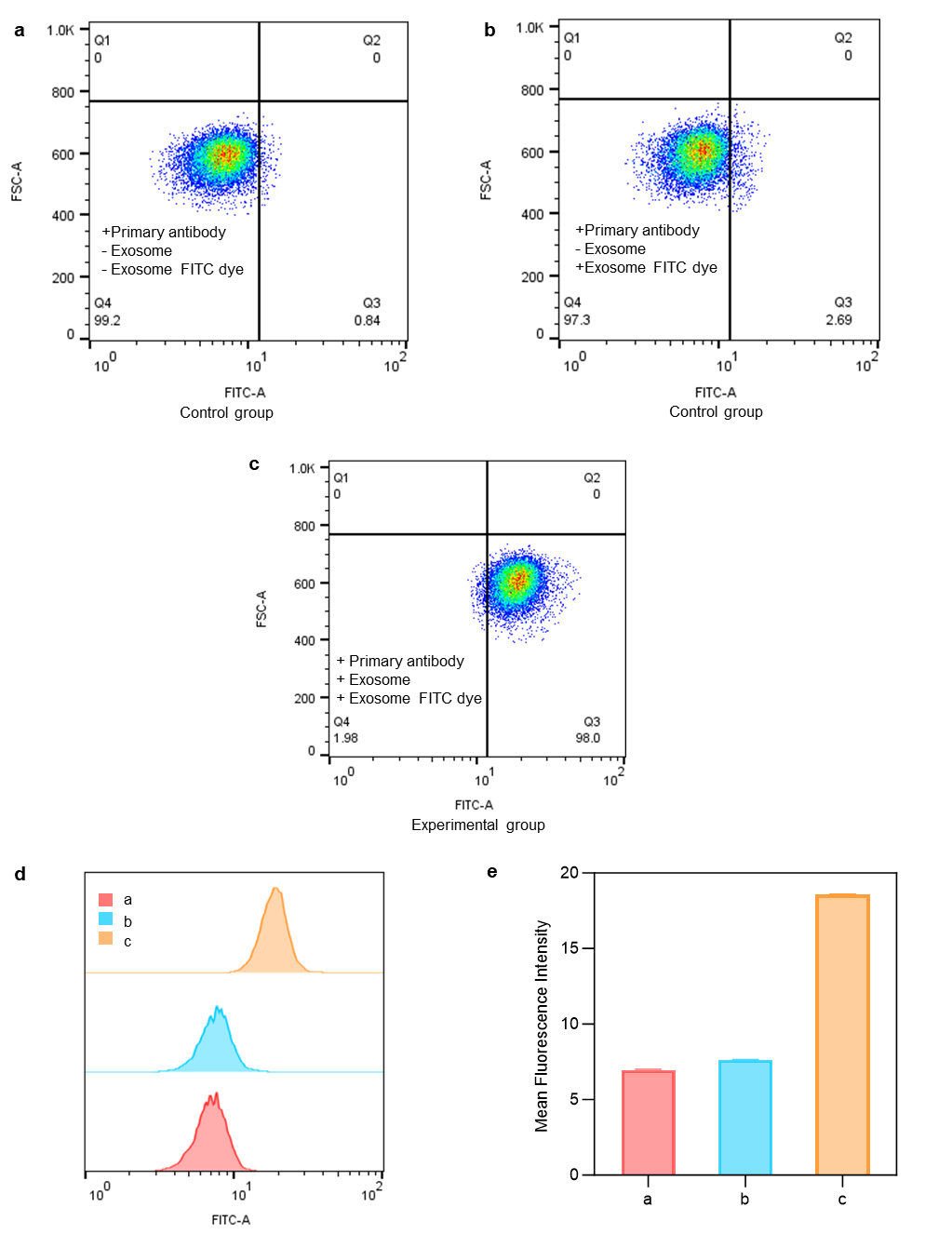


**Figure S5 | Flow cytometer characterization of exosome capture performance of iME beads. a-c,** Flow cytometry dot-plot analysis of blank iME beads **(a)**, iME beads with exosome FITC dye **(b)**, and iME beads captured with FITC labelled exosome **(c)**, showing no cross-reaction between iME beads and exosome FITC dye. **d-e,** Histogram **(d)** and mean **(e)** of the FITC signal intensities of a, b, and c (*n* = 3).

**
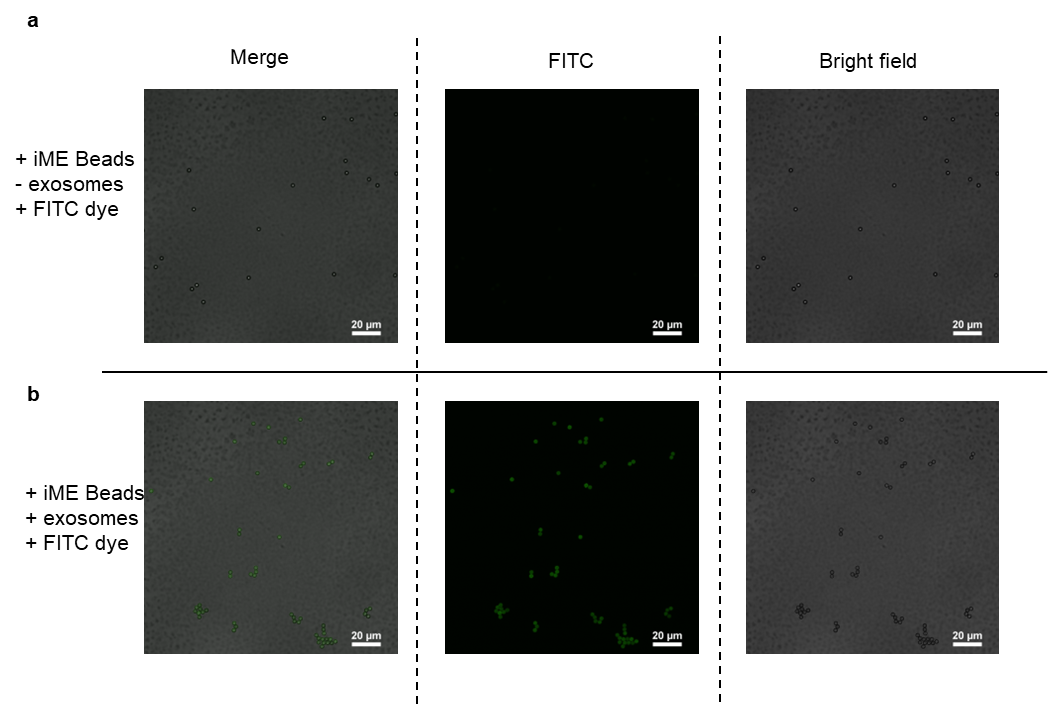
**

**Figure S6 | Fluorescent microscope images of iME beads with FITC exosome dye (a) and iME beads captured with FITC labelled exosomes (b).**


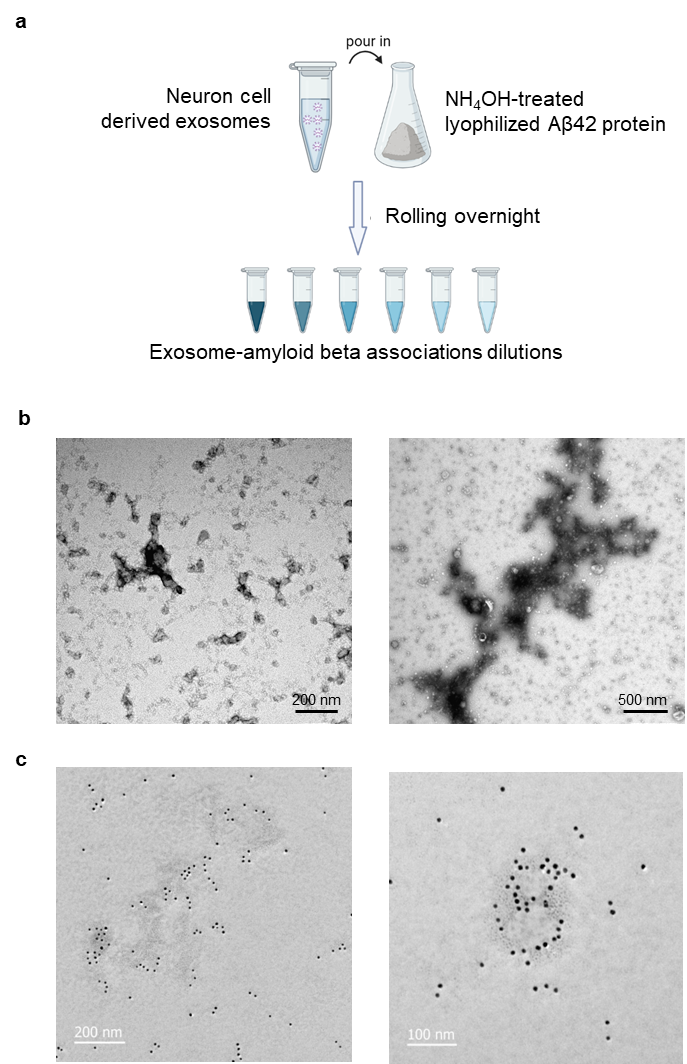


**Figure S7 | Characterization of the prepared Exo-Aβ42 proteins. a,** The schematic illustration of the preparation process. **b,** Transmission electron micrograph images of assembled Aβ42 aggregates (Left) and Exo-Aβ42 protein (Right). **c,** Scanning transmission electron microscopy images of AuNPs (10 nm) immune labelled Aβ42 aggregates (Left) and Exo-Aβ42 protein (Right). Exo-Aβ42 are labeled with AuNPs (10 nm) via a Aβ42-specific antibody. The nanoparticles appear as block dots.

**Figure S8 | Exosome quantification standard curve (*n* = 3).** Four-parameter logistic fitting, *R*² = 0.996.


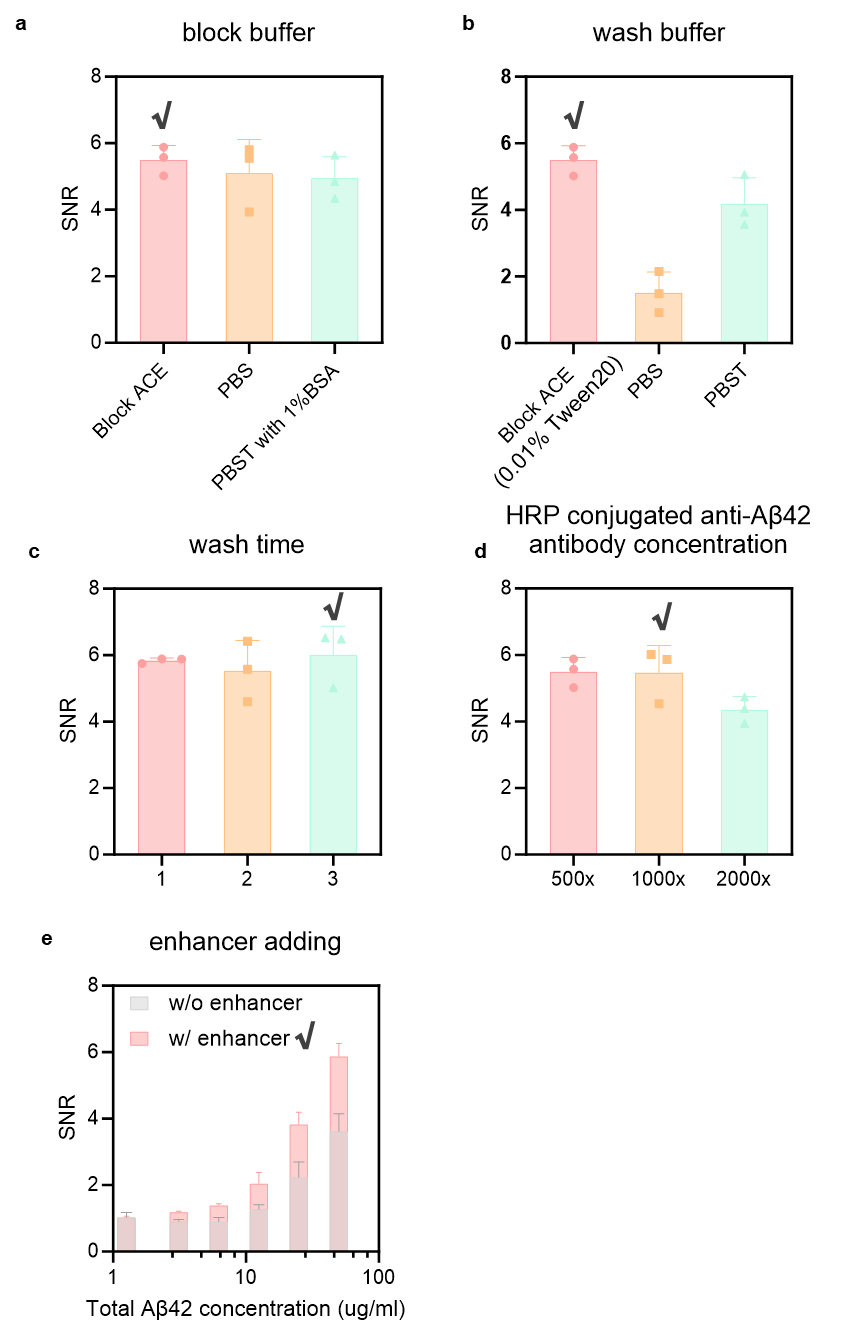


**Figure S9 | Optimization of iME beads-based Exo-Aβ42 identification assay on-chip.** Signal-to-noise ratio of blocking buffer **(a),** washing buffer **(b)**, wash time **(c)**, HRP-conjugated anti-Aβ42 antibody concentration **(d)**, and signal enhancer adding **(e)**.


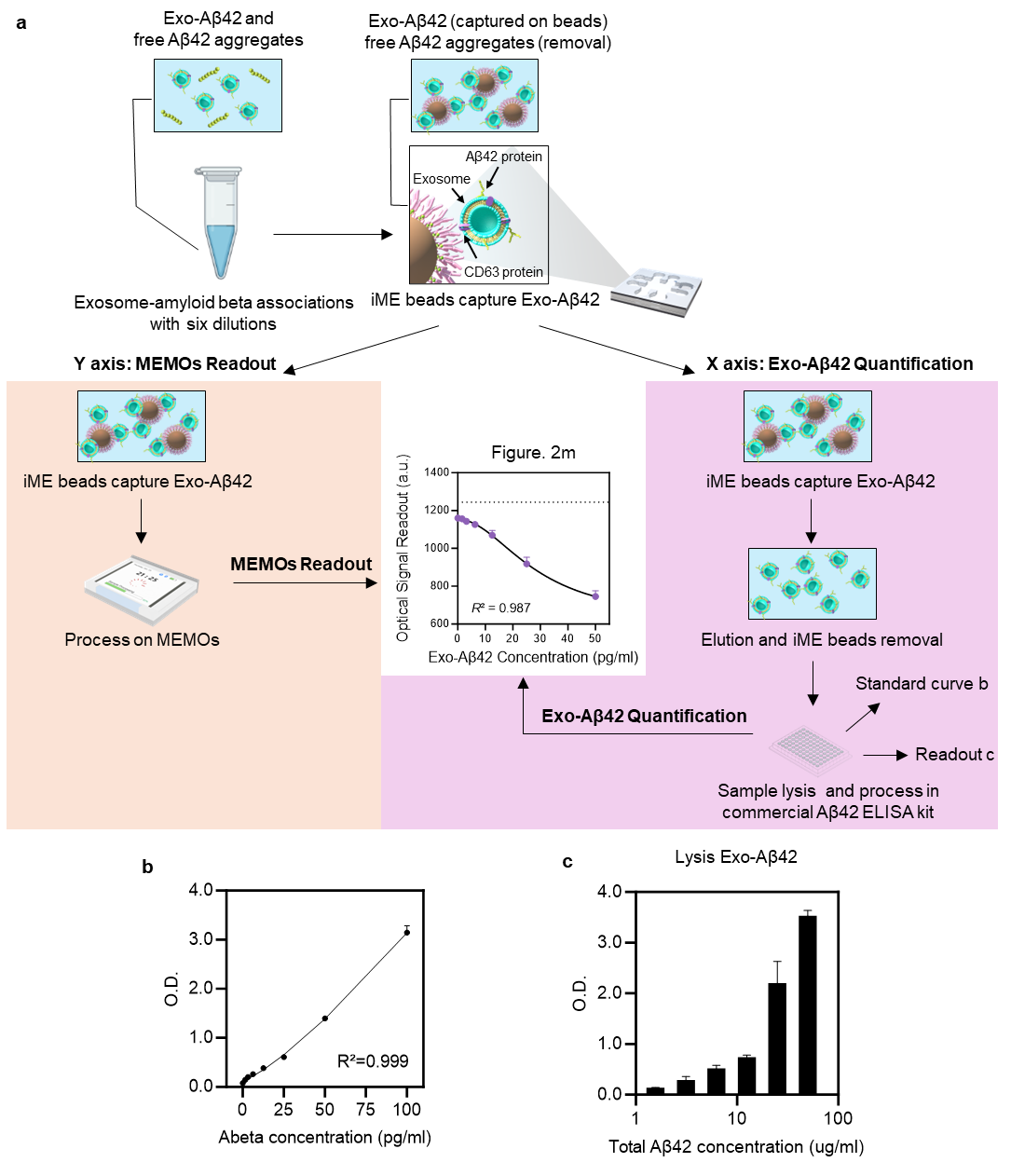


**Figure S10 |** **The corresponding calibration process of MEMOs. a**, The schematic illustration of the corresponding calibration process of MEMOs. Six dilutions of exosome-amyloid beta associations containing both Exo-Aβ42 and free Aβ42 aggregates were prepared. iME beads were used to capture Exo-Aβ42 and isolate them from free Aβ42 aggregates. Then the iME beads captured Exo-Aβ42 proteins were spitted into two parts. One part was processed on MEMOs to obtain the readout, the Y-axis (the optical signal readout of the calibration plot figure.2m). The other part was eluted from beads, lysed and quantified by a commercial Aβ42 ELISA kit to obtain the X-axis (Exo-Aβ42 concentrations of the calibration plot figure.2m). **b**, Commercial Aβ42 protein kit standard curve (*n* = 3). Four-parameter logistic fitting, *R*² = 0.999. **c**, The reference signals of the intermediate process. iME beads captured Exo-Aβ42 markers are lysed and quantified by a commercial Aβ42 kit to characterize the level of Aβ42 that are bounded with exosomes (*n* = 3).

**
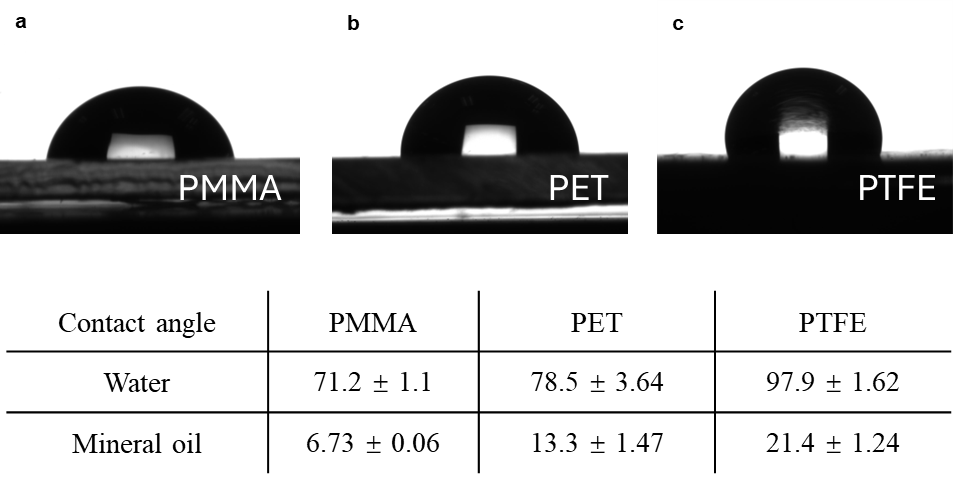
**

**Figure S11 | Contact angles of different substrates. PMMA (a), PET (b), and PTFE (c) (*n* = 3).**

**
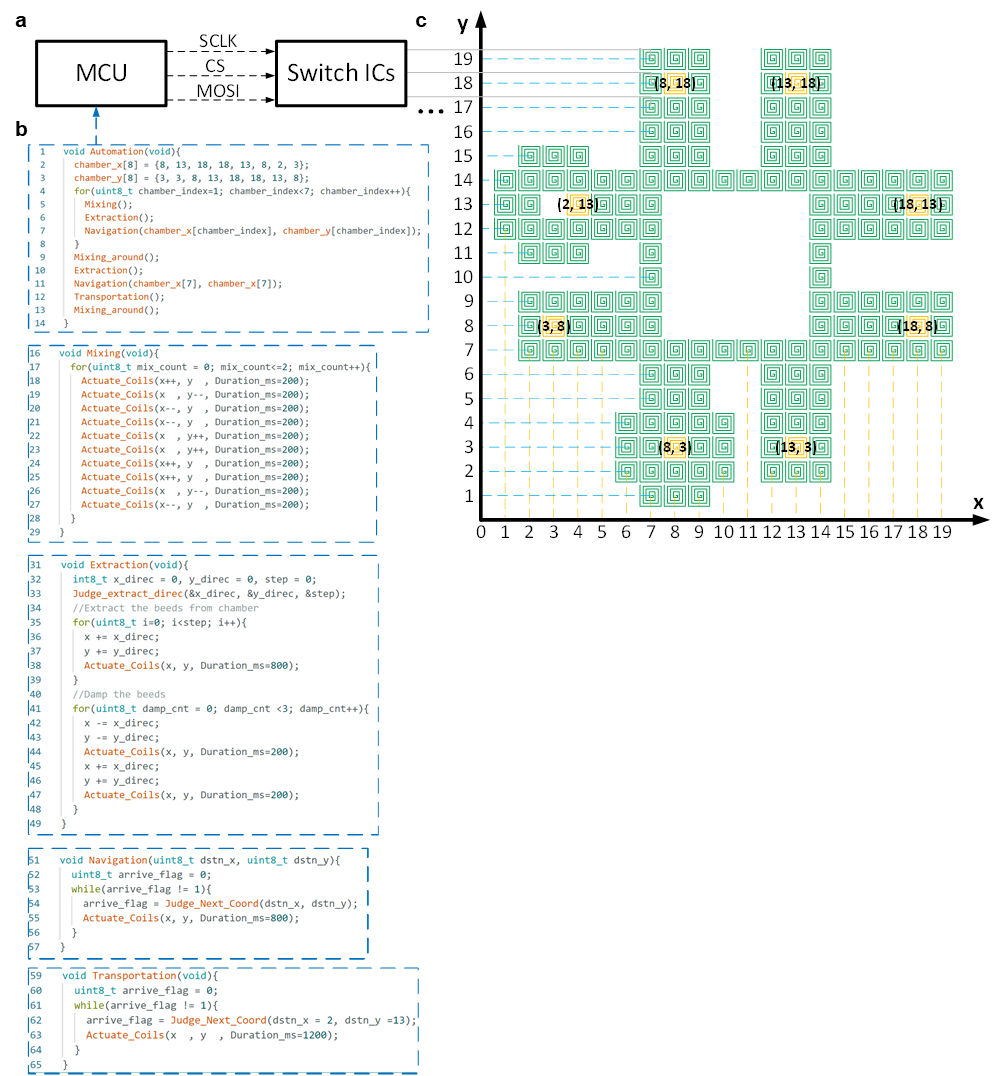
**

**Figure S12 | Administration and implementation of μ-Bead EMchip manipulation at software and hardware levels. a,b,c,** Simplified illustration of the integrated programmed microcontroller **(a)**, which is programmed by different predetermined motion commands **(b)** to activate the magnetic motors and achieve required operations at defined zones **(c)**.

**
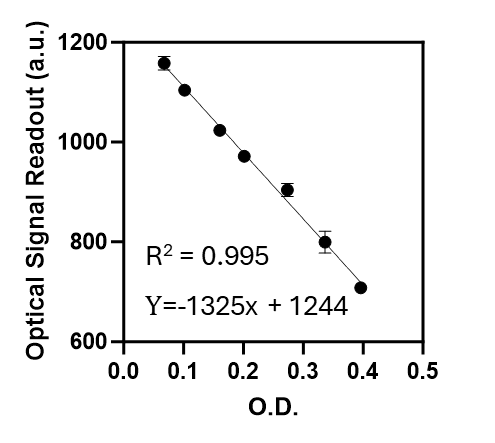
**

**Figure S13 |** **Characterization of optical sensor readout versus standard microplate reader readout.** Error bars indicate SDs (*n* = 3).

**
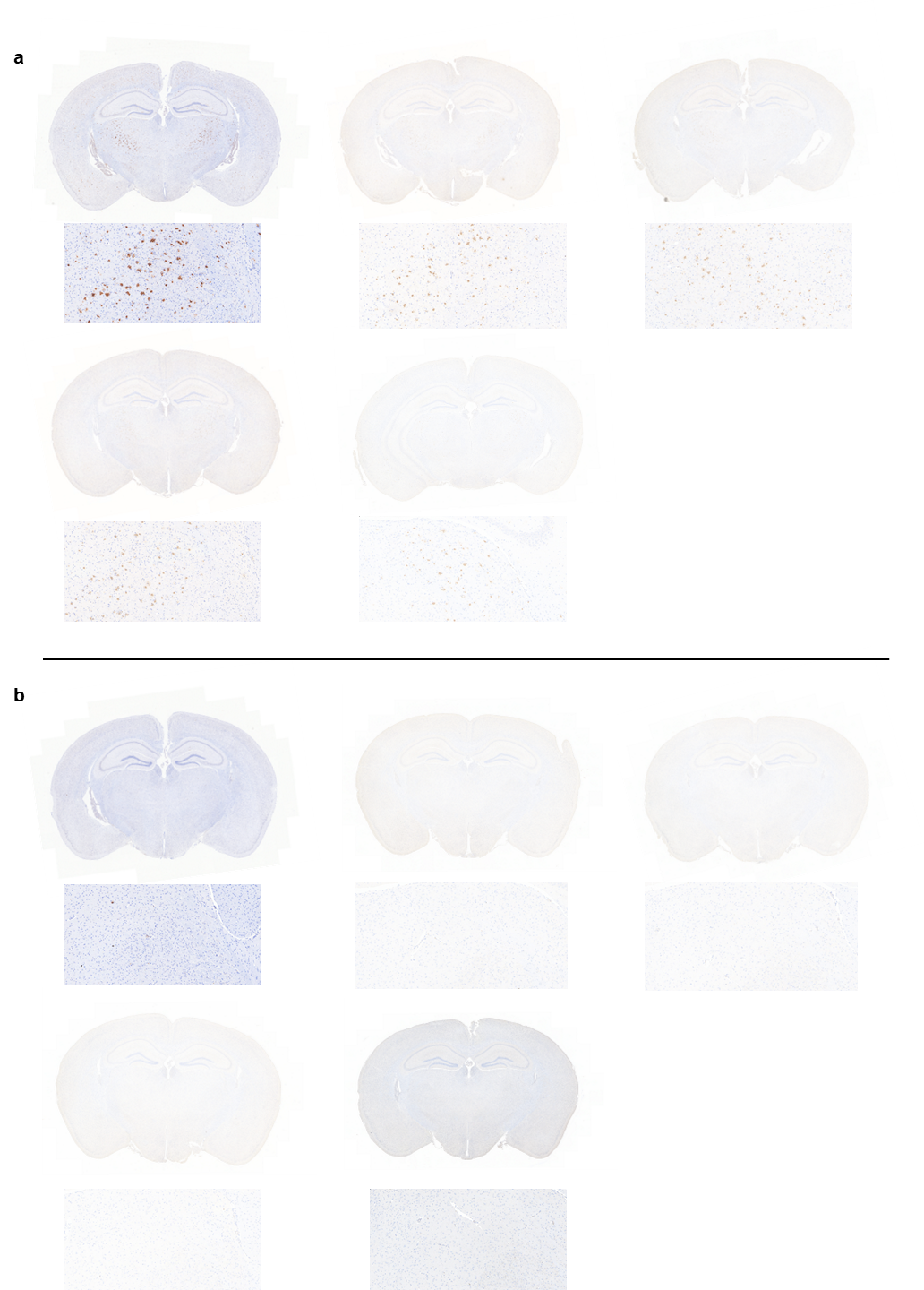
**

**Figure S14 | Immunohistochemical staining of Aβ42 aggregates in the hippocampus of 5xFAD mice (a) (*n* = 5) and C57BL/6J mice (b) (*n* = 5).**

**
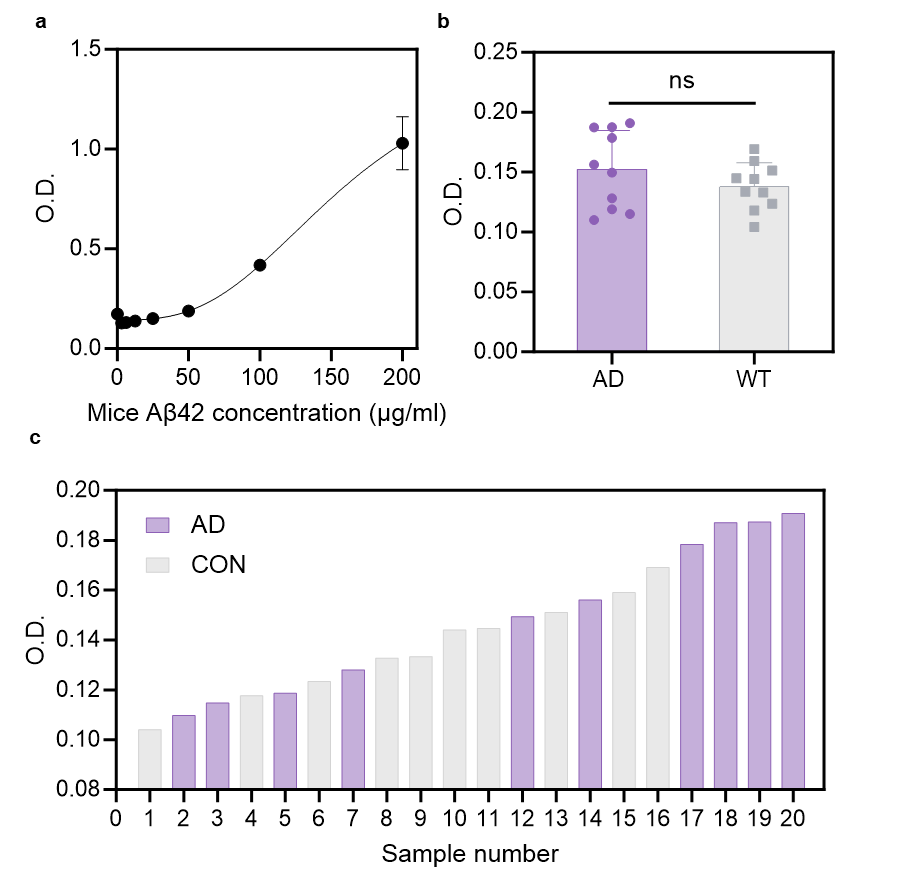
**

**Figure S15 | Commercial ELISA kit characterization of plasma total Aβ42 protein levels in animal samples, including Alzheimer’s mice model: 5xFAD (*n* = 10) and wild type mice model: C57BL/6J (*n* = 10). a,** Commercial Aβ42 protein kit standard curve (*n* = 3). Four-parameter logistic fitting, *R*² = 0.979. **b, c,** Box plots and waterfall plots showing levels of total Aβ42 in plasma measured by ELISA.

**Figure S16 | Receiver operating characteristic (ROC) curve analysis of Exo-Aβ42 levels in plasma measured by MEMOs and Aβ42 levels in plasma tested by ELISA in animal samples.** Alzheimer’s mice model: 5xFAD (*n* = 10) and wild type mice model: C57BL/6J (*n* = 10)

**
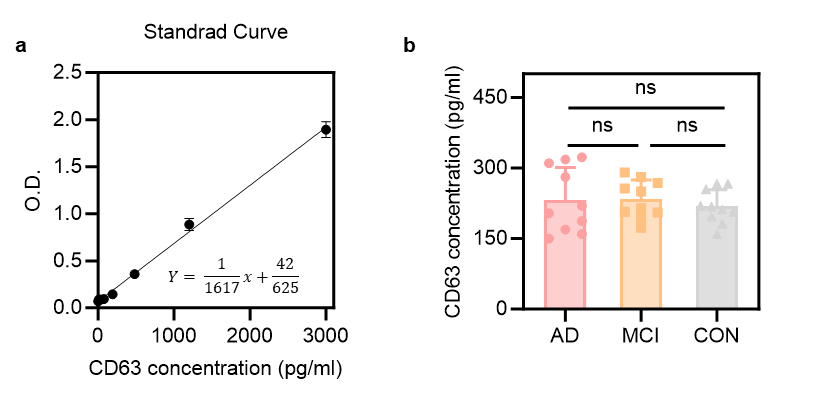
**

**Figure S17 | Characterization of the CD63 protein levels in exosome of human samples. a,** Commercial CD63 protein kit standard curve (*n* = 3). **b,** CD63 protein levels in clinical samples, AD (*n* = 10), MCI (*n* = 10), and CON (*n* = 10). (AD, Alzheimer’s disease patients; MCI, mild cognitive impairment patients; CON, healthy controls)

**
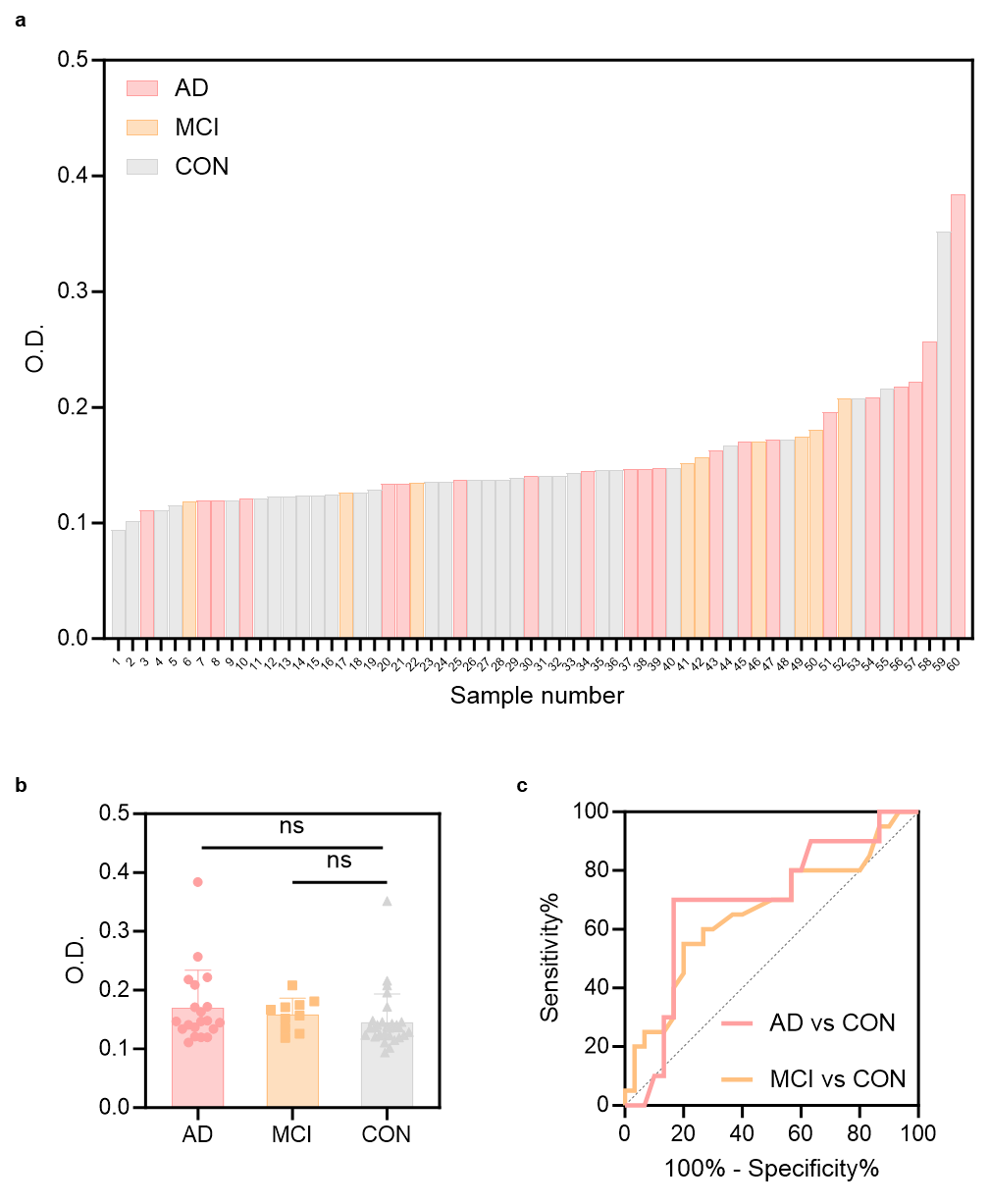
**

**Figure S18 | Characterization of plasma total Aβ42 protein levels in human samples, including AD (*n* = 20), MCI (*n* = 10), and healthy control (*n* = 30). a,** Waterfall plots showing levels of total Aβ42 in plasma measured by ELISA. **b,** Statistical significance analysis of the plasma total Aβ42 protein levels in AD, MCI and CON clinical plasma samples. **c,** Receiver operating characteristic (ROC) curve analysis of total Aβ42 levels in plasma measured by ELISA in clinical samples.

**Table S1 | The list and quantity of components and estimated cost of a MEMOs.**

**Constituent hardware of the System**

| Component | Quantity | Cost (USD) |
| --- | --- | --- |
| PCB board | 1 | 60 |
| Arduino Nano | 1 | 2.95 |
| MAX14662 | 21 | 44.94 |
| LT3092 | 4 | 10 |
| L450R-04 | 1 | 0.68 |
| BH1721 | 1 | 0.48 |
| Resistors | 4 | 1.1 |
| TJ4832T135 | 1 | 16.44 |
| **Total** |  | **136.59** |

**Consumables of the System**

**(Cost per test)**

| Component | | Cost (USD) |
| --- | --- | --- |
| Microfluidic chip | PTFE substrate1 | 0.074 |
|  | PTFE substrate2 | 0.089 |
|  | PET substrate | 0.009 |
|  | Double sided tape | 0.066 |
| Assay reagent | Beads | 1.6 |
|  | Mineral oil | 0.436 |
|  | CD63 antibody | 3.086 |
|  | Aβ42 antibody | 0.322 |
|  | TMB-Stop buffer | 0.032 |
| **Total** |  | **5.714** |

**Table S2 | Service cost comparison between commercialized blood tests and conventional tests in Alzheimer's disease diagnosis** ^[22–24]^.

| **Method** | **Service** | **Launch Date** | **Cost (USD) per test** | **Results Available** | **Accessibility** |
| --- | --- | --- | --- | --- | --- |
| Blood testing | *C_2_N Diagnostics*  Precivity AD^TM^ | Dec-2020 | $1250  (not covered by insurance) | 10 business days | Order by physicians, not public^[25]^ |
|  | *Quest Diagnostics* Quest-AD Detect | May-2022 | $412 (out-of-pocket, may be covered by certain health insurance plans) | 3-10 business days | Order by physicians, not public^[26]^  (since March 12, 2024) |
|  | *Quanterix*  Simoa | Jul-2022 | $300  (not covered by insurance) | 10 business days | Order by physicians, not public^[27]^ |
|  | *C2N Diagnostics* PrecivityAD2TM | Aug-2023 | $1450  (not covered by insurance) | 10 business days | Order by physicians, not public^[28]^ |
|  | *Labcorp*  ATN test | Oct-2023 | $626 (out-of-pocket, may be covered in part by some insurance providers) | 7-10 business days | Order by physicians, not public^[29]^ |
|  | *ALZpath*  ALZpath Dx test | Mar-2024 | $300-$500  (not covered by insurance) | 10 business days | Order by neurologists^[30]^ |
|  | *Eli Lilly*  CertuitAD | Nov-2024 | $195  (not covered by insurance) | 10 business days | Order by neurologists^[31]^ |
| Other testing | Positron emission tomography (PET) |  | range $4000-$5000 | 60 mins testing,  results ready in 24 h | Patient can order^[32]^ |
|  | Cerebrospinal fluid testing (CSF) |  | range $800-$1000 | less than 30 mins procedure,  results ready in about 48-72h | Order by healthcare providers^[33]^ |
|  | Head computed tomography (CT) |  | range $282–$6007 | 60 mins testing,  results ready in 24-48 h | Patient can order^[34]^ |
|  | Brain Magnetic resonance imaging (MRI) |  | range $834–$11524 | 30 mins testing,  results ready in 24-48 h | Order by neurologists and healthcare providers^[34,35]^ |
|  | Neuropsychological examination  (5 hours) |  | range $698–$4165 | 5 h testing,  results ready in a couple of weeks | Order by neurologists and healthcare providers^[34,36]^ |
|  | Neuropsychological examination  (8 hours) |  | range $1104–$6657 | 8 h testing,  results ready in a couple of weeks | Order by neurologists and healthcare providers^[34,37]^ |

**References for Supplementary Information**

[1] T. Kong, R. Brien, Z. Njus, U. Kalwa, and S. Pandey, “Motorized actuation system to perform droplet operations on printed plastic sheets,” Lab Chip, vol. 16, no. 10, pp. 1861–1872, 2016, doi: 10.1039/C6LC00176A.

[2] Z. Long, A. M. Shetty, M. J. Solomon, and R. G. Larson, “Fundamentals of magnet-actuated droplet manipulation on an open hydrophobic surface,” Lab Chip, vol. 9, no. 11, pp. 1567–1575, 2009, doi: 10.1039/B819818G.

[3] G. Cai, Z. Yang, Y.C. Chen, Y. Huang, L. Liang, S. Feng, J. Zhao, “Magnetic Bead Manipulation in Microfluidic Chips for Biological Application,” Cyborg and Bionic Systems, vol. 4, p. 0023, 2023, doi: 10.34133/CBSYSTEMS.0023.

[4] N. Pamme, “Magnetism and microfluidics,” Lab Chip, vol. 6, no. 1, pp. 24–38, 2006, doi: 10.1039/B513005K.

[5] H. Y. Kim, H. J. Lee, and B. H. Kang, “Sliding of Liquid Drops Down an Inclined Solid Surface,” J Colloid Interface Sci, vol. 247, no. 2, pp. 372–380, 2002, doi: 10.1006/JCIS.2001.8156.

[6] A. Li, H. Li, Z. Li, Z. Zhao, K. Li, M. Li, Y. Song, “Programmable droplet manipulation by a magnetic-actuated robot,” Sci Adv, vol. 6, no. 7, eaay5808, 2020, doi: 10.1126/SCIADV.AAY5808.

[7] Y. Yang and Y. Zeng, “Microfluidic communicating vessel chip for expedited and automated immunomagnetic assays,” Lab Chip, vol. 18, no. 24, pp. 3830–3839, 2018, doi: 10.1039/C8LC00927A.

[8] S. Hosseini, P. Vázquez-Villegas, M. Rito-Palomares, and S. O. Martinez-Chapa, “Advantages, Disadvantages and Modifications of Conventional ELISA,” *Enzyme-linked Immunosorbent Assay (ELISA),* Springer Singapore, ISBN 978-981-10-6765-5, pp. 67–115, 2018, doi: 10.1007/978-981-10-6766-2_5.

[9] C. Zhou, Z. Fang, C. Zhao, X. Mai, S. Emami, A.Y. Taha, G. Sun, T. Pan, “Sample-to-Answer Robotic ELISA,” Anal Chem, vol. 93, no. 33, p. 11424, 2021, doi: 10.1021/ACS.ANALCHEM.1C01231.

[10] D. H. Wilson, D.M. Rissin, C.W. Kan, D.R. Fournier, T. Piech, T.G. Campbell, R.E. Meyer, M.W. Fishburn, C. Cabrera, P.P. Patel, E. Frew, “The Simoa HD-1 Analyzer: A Novel Fully Automated Digital Immunoassay Analyzer with Single-Molecule Sensitivity and Multiplexing,” SLAS Technol, vol. 21, no. 4, pp. 533–547, 2016, doi: 10.1177/2211068215589580.

[11] N. Dilsiz, “A comprehensive review on recent advances in exosome isolation and characterization: Toward clinical applications,” Transl Oncol, vol. 50, p. 102121, 2024, doi: 10.1016/J.TRANON.2024.102121.

[12] M. Wu, Y. Ouyang, Z. Wang, R. Zhang, P.-H. Huang, C. Chen, H. Li, P. Li, D. Quinn, M. Dao, S. Suresh, Y. Sadovsky, T. J. Huang, “Isolation of exosomes from whole blood by integrating acoustics and microfluidics,” Proc Natl Acad Sci USA, vol. 114, no. 40, pp. 10584–10589, 2017, doi: 10.1073/PNAS.1709210114.

[13] E. Beitello, K. Osei, T. Kobulnicky, F. Breausche, J. A. Friesen, and J. D. Driskell, “Oriented Surface Immobilization of Antibodies Using Enzyme-Mediated Site-Specific Biotinylation for Enhanced Antigen-Binding Capacity,” Langmuir, vol. 41, no. 16, pp. 10576–10585, 2025, doi: 10.1021/ACS.LANGMUIR.5C00656.

[14] Y. Zhou, W. Hu, B. Peng, and Y. Liu, “Biomarker binding on an antibody-functionalized biosensor surface: The influence of surface properties, electric field, and coating density,” Journal of Physical Chemistry C, vol. 118, no. 26, pp. 14586–14594, 2014, doi: 10.1021/JP501885P.

[15] M. Villa, J. Sans, M. Arnau, F. Estrany, E. Armelin, T. Ramón, V. Cebrián, Ó. Ahumada, C. Alemán, “Strategy to optimize the surface functionalization of immunosensors and improve their detection efficiency,” Appl Surf Sci, vol. 705, p. 163529, 2025, doi: 10.1016/J.APSUSC.2025.163529.

[16] J. D. Hirsch and R. P. Haugland, “Conjugation of Antibodies to Biotin,” Methods Mol Biol, vol. 295, pp. 135–154, 2005, doi: 10.1385/1-59259-873-0:135.

[17] M. Conrad, G. Proll, E. Builes-Münden, A. Dietzel, S. Wagner, and G. Gauglitz, “Tools to compare antibody gold nanoparticle conjugates for a small molecule immunoassay,” Microchimica Acta, vol. 190, no. 2, pp. 1–12, 2023, doi: 10.1007/S00604-023-05637-X.

[18] S. P. Kent, K. H. Ryan, and A. L. Siegel, “Steric hindrance as a factor in the reaction of labeled antibody with cell surface antigenic determinants.,” Journal of Histochemistry & Cytochemistry, vol. 26, no. 8, pp. 618–621, 1978, doi: 10.1177/26.8.357645.

[19] F. Li, H. Xu, P. Sun, Z. Hu, and Z. P. Aguilar, “Size effects of magnetic beads in circulating tumour cells magnetic capture based on streptavidin–biotin complexation,” IET Nanobiotechnol, vol. 13, no. 1, p. 6, 2018, doi: 10.1049/IET-NBT.2018.5104.

[20] D. Xu and S. V. Wegner, “Multifunctional streptavidin–biotin conjugates with precise stoichiometries,” Chem Sci, vol. 11, no. 17, pp. 4422–4429, 2020, doi: 10.1039/D0SC01589J.

[21] H. M. Yang, R. M. Bao, C. M. Yu, Y. N. Lv, W. F. Zhang, and J. B. Tang, “Fc-specific biotinylation of antibody using an engineered photoactivatable Z–Biotin and its biosensing application,” Anal Chim Acta, vol. 949, pp. 76–82, 2017, doi: 10.1016/J.ACA.2016.10.039.

[22] “Alzheimer’s Blood Tests: How Do They Work and Should You Request One?” Accessed: Mar. 05, 2025. Available: <https://www.brightfocus.org/resource/alzheimers-blood-tests-how-do-they-work-and-should-you-request-one/> (2025).

[23] “Your Guide to Alzheimer’s Blood Tests: Pricing, Accuracy, and Where to Get One | Being Patient.” Accessed: Mar. 05, 2025. Available: <https://www.beingpatient.com/how-much-is-an-alzheimers-blood-test/> (2025).

[24] K. Noda, Y. Lim, R. Goto, S. Sengoku, and K. Kodama, “Cost-effectiveness comparison between blood biomarkers and conventional tests in Alzheimer’s disease diagnosis,” Drug Discov Today, vol. 29, no. 3, p. 103911, Mar. 2024, doi: 10.1016/J.DRUDIS.2024.103911.

[25] “PrecivityAD®.” Accessed: Mar. 05, 2025. Available: <https://precivityad.com/>.

[26] “Dementia Testing | Quest Diagnostics.” Accessed: Mar. 05, 2025. Available: <https://www.questdiagnostics.com/healthcare-professionals/about-our-tests/neurological-disorders/alzheimers>.

[27] “Simoa® Technology | Quanterix.” Accessed: Mar. 05, 2025. Available: <https://www.quanterix.com/simoa-technology/>.

[28] “About PrecivityADTM — PrecivityAD®.” Accessed: Mar. 05, 2025. Available: <https://precivityad.com/precivityad2-hcp>.

[29] “484400: ATN Profile | Labcorp.” Accessed: Mar. 05, 2025. Available: <https://www.labcorp.com/tests/484400/atn-profile>.

[30] M. M. Paczynski and G. S. Day, “Alzheimer Disease Biomarkers in Clinical Practice: A Blood-Based Diagnostic Revolution,” J Prim Care Community Health, vol. 13, 2022, doi: 10.1177/21501319221141178.

[31] “Blood Test to Help Detect Alzheimer’s Disease Pathology | CertuitAD®.” Accessed: Mar. 05, 2025. Available: <https://www.certuitad.com/>.

[32] “PET Scan: What It Is, Types, Purpose, Procedure & Results.” Accessed: Mar. 05, 2025. Available: <https://my.clevelandclinic.org/health/diagnostics/10123-pet-scan>.

[33] “Cerebrospinal Fluid (CSF) Testing - Testing.com.” Accessed: Mar. 05, 2025. Available: <https://www.testing.com/tests/cerebrospinal-fluid-csf-testing/>.

[34] N. Kurniadi, J. Davis, K. Kitchen-Andren, C. Mullen, S. Rolin, “A-239 Cost of Neuropsychological Evaluation Comparable to Neuroimaging in the Eastern USA,” Archives of Clinical Neuropsychology, vol. 35, no. 6, pp. 1034–1034, 2020, doi: 10.1093/ARCLIN/ACAA068.239.

[35] “Brain MRI: What It Is, Purpose, Procedure & Results.” Accessed: Mar. 05, 2025. Available: <https://my.clevelandclinic.org/health/diagnostics/22966-brain-mri>.

[36] “Neuropsychological Testing: What It Is, Purpose & Procedure.” Accessed: Mar. 05, 2025. Available: <https://my.clevelandclinic.org/health/diagnostics/4893-neuropsychological-testing-and-assessment>.

[37] “Neuropsychological Testing: What It Is, Purpose & Procedure.” Accessed: Mar. 05, 2025. Available: <https://my.clevelandclinic.org/health/diagnostics/4893-neuropsychological-testing-and-assessment>.
